# Stability Analysis and Identification of Superior Hybrids in Pearl Millet [*Pennisetum glaucum* (L.) R. Br.] Using the Multi Trait Stability Index

**DOI:** 10.3390/plants13081101

**Published:** 2024-04-15

**Authors:** Vikas Khandelwal, Rumit Patel, Khushwant B. Choudhary, S. B. Pawar, M. S. Patel, K. Iyanar, K. D. Mungra, Sushil Kumar, C. Tara Satyavathi

**Affiliations:** 1 ICAR-All India Coordinated Research Project on Pearl Millet, AU, Jodhpur 342 304, India; 2 Department of Agricultural Biotechnology, Anand Agricultural University, Anand 388 110, India; rumitpatel01@gmail.com (R.P.); sushil254386@yahoo.com (S.K.); 3 ICAR-Central Arid Zone Research Institute (CAZRI), Jodhpur 342 003, India; khushwantchoudhary1@gmail.com; 4 National Agricultural Research Project, Vasantrao Naik Marathwada Krishi Vidyapeeth, Aurangabad 431 005, India; drsuryakantpawar@gmail.com; 5 Centre for Crop Improvement, Sardarkrushinagar Dantiwada Agricultural University, Banaskantha 385 506, India; mspatel1966@gmail.com; 6 Department of Millets, Tamil Nadu Agricultural University, Coimbatore 641 003, India; iyanar.k@tnau.ac.in; 7 Pearl Millet Research Station, Junagadh Agricultural University, Junagadh 362 001, India; kdmungra@jau.in; 8 Indian Institute of Millets Research, Hyderabad 500 030, India; aicpmip@gmail.com

**Keywords:** pearl millet, stability, MTSI, hybrids, G × E interaction

## Abstract

Pearl millet stands as an important staple food and feed for arid and semi-arid regions of India and South Africa. It is also a quick supplier of important micronutrients like Fe and Zn via grain to combat micronutrient deficiencies among people in developing countries. India has notably spearheaded advancements in pearl millet production and productivity through the All India Coordinated Pearl Millet Improvement Project. There were 21 hybrids evaluated over arid and semi-arid ecologies of the western and southern regions of India. AMMI and GGE biplot models were adopted to recommend a specific hybrid for the particular locality. A joint analysis of variation indicated a significant genotype–environment interaction for most of the agronomical and grain micronutrient parameters. Pearson’s correlation values dissected the significant and positive correlation among agronomic traits and the negative correlation with grain micronutrient traits. GGE biplot analysis recommended the SHT 106 as a dual-purpose hybrid and SHT 115 as a biofortified hybrid for the grain’s Fe and Zn content. SHT 110 and SHT 108 were selected as stable and high grain yield-producing hybrids across all environments and specifically for E1, E2, and E4 as per the Which-Won-Where and What biplot. SHT 109 and SHT 103 hybrids were stable and high dry fodder yield-producing hybrids across all environments. In this study, the Multi-Trait Stability Index (MTSI) was employed to select the most stable and high-performing hybrids for all traits. It selected SHT 120, SHT 106, and SHT 104 for stability and great performance across all environments. These findings underscored the significance of tailored hybrid recommendations and the potential of pearl millet in addressing both food security and malnutrition challenges in various agro-ecological regions.

## 1. Introduction

Pearl millet [*Pennisetum glaucum* (L.) R. Br.] is a coarse cereal crop belonging to the Poaceae family [1]. It is cross-pollinated in nature due to its protogynous blooming pattern where the stigma matures earlier than the anthers. This cross-pollinating behavior creates great variability in the genome by random matting and crossing over between homologous chromosomes. It has a genome size of 1.79 GB, which is organized into seven linkage groups. Pearl millet is largely cultivated in the drought-affected areas of (semi)arid conditions of Indian and African regions. It is very productive in those areas because it has a high biomass-producing ability due to the C4 (Kranz anatomy) mechanism for carbon fixation [2,3]. In this mechanism, mesophyll cells are gathered around the bundle-sheath cells in a ring-like fashion in which the initial carbon fixation takes place in the mesophyll cells and the Calvin cycle takes place in the bundle-sheath cells. This provides an advantage against photorespiration resulting in high productivity and good development [4].

Worldwide, half of all millet production is composed of pearl millet. It is primarily cultivated in Africa and India, covering 28 million hectares. It serves as a staple diet for millions of people living in arid and semi-arid areas due to its vital nutritional qualities like protein, carbohydrates, fat, and minerals. It contains 2.16–2.78 g per 100 g of total sugars, 56.05–73.37% starch, 1.5–9.9% fat, 6.40–24.25% protein, 30.1–75.7 mg kg^−1^ of iron, 24.5–64.8 mg kg^−1^ of zinc, 185.0–363.0 mg per 100 g of phosphorous, and 84–445 mg per 100 g of total carotenoids [5]. Additionally, it has antioxidants such as phenols and phytic acid that help to prevent metabolic diseases, cancer, heart disease, and aging. Millions of individuals suffer from “hidden hunger”, sometimes referred to as micronutrient deficiencies around the world. Pearl millet is a rich source of iron and zinc that can help combat micronutrient deficiency through genetic biofortification, while its biological yield is utilized as animal feed. India is a leading harvester of pearl millet with 11 million tonnes per year, which is around 36% of the global production. From a 6.93-million-hectare area with 1243 kg ha^−1^ of productivity, India collected 8.61 million tonnes of pearl millet grains in 2020 [6].

This record production is attributed to the adoption of hybrids. However, before 1940, farmers relied only on the open-pollinated varieties (OPVs). In early 1940, the Indian Council of Agricultural Research (ICAR) initiated sporadic efforts for pearl millet improvement. To channel the improvement, ICAR started the All India Co-ordinated Millet Improvement Project (AICMIP) in 1965. Currently, it is separated from other millets and the project is now known as the All India Coordinated Pearl Millet Improvement Project (AICPMIP). Initially, emphasis was given to research on the development of high grain yield-producing and broad adaptive open-pollinated varieties through simple plant selection and mass selection methods. Over time, the focus has broadened from high-yielding cultivars to cultivars with great biotic and abiotic stress tolerance capacity with micronutrient biofortification.

Burton [7] reported 70–80% cross-pollination in pearl millet due to its protogynous flowering behavior with a wind-born pollination mechanism. It fulfills the essential biological requirement for hybrid development. Moreover, pearl millet has commercially exploitable cytoplasmic-nuclear male sterility, which leads to the development of hybrid cultivars. Additionally, the cross-pollination mechanism adds the advantage of developing synthetic and composite open-pollinated cultivars. The developed hybrids and OPVs should exhibit stability in performance across various locations over different years [8]. OPVs are genetically heterogeneous and heterozygous, which leads to variable expression across the field. On the other hand, single cross hybrids are developed using pure inbreds, making them genetically heterozygous but homogeneous. This provides stable performances across the field. To check the stable performance of newly developed hybrids, AICPMIP-ICAR tested them in multiple locations through their network. They adopted various stability parameters to interpret genotype–environment interactions and recommend hybrids suitable for specific localities. Memon et al. [9] indicated various stability methods for ranking genotypes and identifying suitable environments. For multi-environment trial (MET) data analysis, the Additive Main effects and Multiplicative Interaction (AMMI) model and GGE biplots are commonly utilized. However, the AMMI model does not consider the genotypic effect. Yan et al. [10] resolved this problem by developing the Genotype × Genotype × Environment (GGE) biplot technique, which considers both a genotype’s main effects and GEI effects. Thus, the GGE biplot model is considered a useful technique for determining ideal genotypes and testing conditions [11].

The theoretical foundation of MTSI was proposed by Olivoto et al. [12] for the selection of high-yielding and stable genotypes for multiple traits based on multi-environment trait data considering random and fixed-effect models. It is calculated based on the ideal genotype–ideotype distance using factor analysis. This selection index allows for the selection of stable genotypes with a positive selection differential for traits intended to be increased and a negative selection differential for traits intended be to decreased. This also combines the average performance and stability of the traits, aiding breeders in simultaneous selection for yield as well as stability. Based on the above discussion, we addressed the following objectives: (1) multi-environment trait data were subjected to stability analysis to identify hybrids with general stability; (2) identify and recommend hybrids with specific stability and specific features; (3) selection of the most stable and best-performing hybrids for multiple traits.

## 2. Results and Discussion

### 2.1. ANOVA and Mean Performance

**Figure 1:**
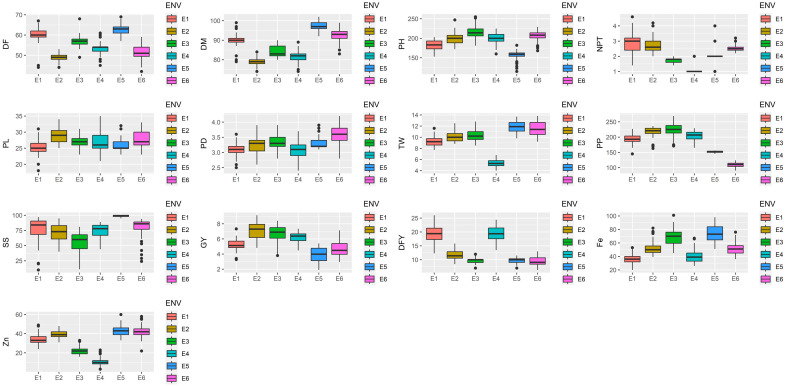
Box plots showing mean performances of the studied traits across all environments during Summer-2021. DF: days to 50% flowering, DM: days to maturity, PH: plant height, NPT: number of productive tillers per plant, PL: panicle length, PD: panicle diameter, TSW:1000 seed weight, PP: plant population at harvest, SS: seed set percentage under bagging condition, GY: grain yield, DFY: dry fodder yield, Fe: grain iron content, Zn: grain zinc content, ENV: environments, E1: Mandor, E2: Jamnagar, E3: S. K. Nagar, E4: Ahmedabad, E5: NARP, Aurangabad, E6: Coimbatore.

A combined ANOVA disclosed a significant difference among all hybrids for the studied traits. PL exhibited the highest variance for GEI at 31.73% among all traits, followed by PD at 28.54%, seed set under bagging conditions at 24.54%, and GY at 21.42%, as detailed in Table 1. As indicated by the genotype mean square, hybrids displayed significant variation for the studied traits evaluated across the locations. However, it was observed that locations contributed the highest variation for all traits. (Table 1). The maximum variance for locations among all variables was 96.04% for plant population at harvest, which indicated the different survival of hybrids across changing environments. E3 (220.67) showed the highest plant population while E6 (109.16) showed the lowest plant population across all environments (Figure 1 and Table S3). According to Table 1, GEI (3.13%) did not play any significant role in plant population at harvest, indicating that all the locations are preferred for pearl millet cultivation. Zinc content showed 92.41% environmental variance followed by 87.51% for dry fodder yield per plant, 86.64% for test weight, and 86.56% for days to maturity. In the present investigation, most of the characters indicated considerable genotype–environment interactions (GEIs) in a joint analysis of variance except PTPP and plant population at harvest, indicating that the stability of hybrids should be investigated further for important traits like grain yield per net plot, dry fodder yield per net plot, and Fe and Zn content. Gangashetty et al. [13] found significant genotypic and environmental effects for PH, PL, and mineral content. Sodhaparmar et al. [14] recorded significant GEI effects for most of the traits except days to 50% flowering, effective tillers per plant, ear head length, and fodder yield. Sanjana Reddy et al. [15] also reported significant results for all the agronomic traits under study. Based on the significant results of a combined ANOVA, Gangashetty et al. [13] and Sanjana Reddy et al. [15] suggested dissecting stability using the AMMI model. Mean grain yield per plant was 5.64 kg per net plot across the environments with a range of 3.91 (E5) to 7.11 kg per net plot (E2) (Table 1 and Table S3). Dry fodder yield ranged from 9.60 (E3) to 19.46 (E1) with a 13.24 kg per net plot across all environments. Similarly, micronutrient Fe ranged from 36.14 (E1) to 68.79 mg kg^−1^ with a mean of 53.80 mg kg^−1^ across environments. Likewise, Table 1 shows the means of the other characters studied across environments, which were 55.49 (DF), 87.42 (DM), 193.04 (PH), 2.17 (PTPP), 26.92 (PL), 3.27 (PD), 9.71 (TW), 183.26 (PP), 75.36 (SS%), and 31.93 (Zn), and the boxplot (Figure 1) depicts the mean of the characters of individuals for each environment. Similarly, Gangashettty et al. [13] reported 43.32 to 63.15 days for 50% days to flowering among 20 genotypes. They also found the same trend for other agronomic traits and all the mineral contents. Sanjana Reddy et al. [15] found similar results for PH, PL, Fe, and Zn content in all locations under study.

### 2.2. GEI Analysis

AMMI analysis showed a significant difference in the additive component of the total sum of squares attributed to genotypic influence, GEI effect, and environment except for plant population at harvest and PTPP (Table 1). This finding indicated that changes in environmental conditions had a substantial influence on the majority of traits. Gangashetty et al. [13] reported the highest genotypic variance for PH, PL, DF, and GY. They also identified comparable interaction effects between location and genotype, demonstrating significant differences at *p* < 0.001 for DF, PH, PL, and GY. Asungre et al. [16] reported similar results in most of the traits while assessing the stability and adaptability of pearl millet hybrids for GY and grain mineral content in Ghana using the AMMI model. The AMMI model dissected the GEI effect into five interaction components commonly referred to as the multiplicative effect (Table 2). These were divulged through principal component analysis [17], as depicted in Table 2. According to Yan et al. [10], strong GEI impacts reduce the gain for quantitative characteristics such as GY and dry fodder yield. However, in the present study, the proportion of GEI was found to be medium to low. This indicated that most of the traits can improve in a wide range of environments.

### 2.3. AMMI Biplot

**Figure 2:**
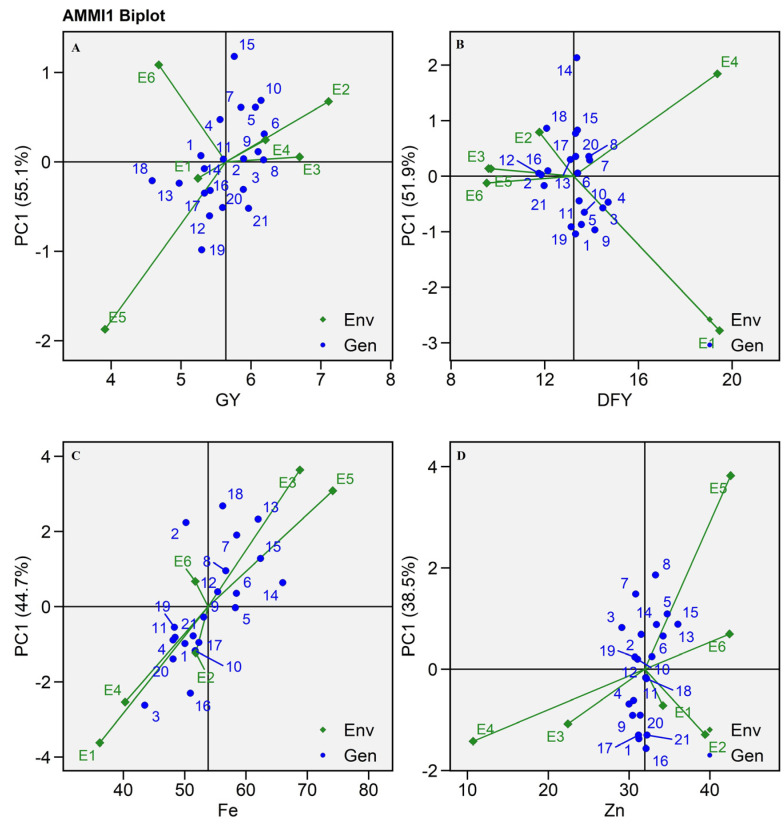
AMMI I biplot of 21 pearl millet hybrids (Blue text) evaluated in six environments (Green text) during summer-2021 (**A**) GY: Grain yield, (**B**) DFY: Dry fodder yield, (**C**) Fe: Iron content, (**D**) Zn: Zinc content. PC: principal component.

Various biplots were employed to illustrate the potential of hybrids across different traits. In the current study, grain yield, dry fodder yield, and mineral content were analyzed using different biplots. The AMMI I biplot was generated by displaying the mean of the trait across the environments on the *X*-axis, referred to as the main effects [17]. The *Y*-axis represents the IPCA I score, capturing multiplicative effects (Figure 2). The standard interpretation of this biplot is that if a genotype or an environment has a PC score of nearly zero, this indicates a minimum interaction, while genotype and environment PC scores with a common sign suggest a positive interaction between them [18,19,20].

A significant GEI was observed for grain yield, dry fodder yield, and mineral content with values of 21.42%, 9.48%, 13.57%, and 5.81% variance, respectively (Table 1). This interaction was further subdivided into five interactive PCs. Among all five components, the first two components explained the maximum interactive variance (66.0 to 79%). This suggested the IPCA I axis contributed to the majority of the variance (Table 2 and Figure 2). In the case of grain yield (GY), environments E1, E6, and E5 positioned on the left side of the mean yield line indicated less productive environments. Conversely, the remaining environments, namely E2, E3, and E4 positioned on the right side of the mean yield line, suggested more productive environments as per Pawar et al. [21]. Among all the productive environments, E4 was found to be the least changing environment because it was positioned close to the origin with the shortest vector, while the rest of the environments with extended vectors suggested highly interactive environments.

Similarly, E1 and E4 were found to be the most productive environments for dry fodder yield as they showed higher DFY production compared to other environments, but these two environments were highly interactive with hybrids as they were situated far from the origin with the longest vector (Table S3 and Figure 2). However, in the case of the micronutrient content, the opposite trend was observed. For Fe and Zn content, E5 was found to be the most interactive environment and most productive. This environment was found to be least productive for grain yield and dry fodder yield. This result indicated that hybrids accumulate micronutrients in higher concentrations when they are grown in a less productive environment (Figure 2).

Asungre et al. [16] validated the current findings by revealing a 50.7% variation in the IPCA I axis in pearl millet. In this study, SHT 102, SHT 108, and SHT 109 had near-zero IPCA I value with high means for grain yield; hence, they were located on the right side of the overall mean line (Figure 2A). Furthermore, they were characterized by minimal interactions with the environment, evident by their placement along the IPCA I line in the AMMI I biplot (Table 3 and Figure 2A). Similarly, SHT 106 was the least interactive for dry fodder yield (Table S5 and Figure 2B). SHT 105 was the least interactive and most productive hybrid for Fe content, while none of the hybrids were displayed on the IPCA I line for Zn content (Table S3, Figure 2C,D).

**Figure 3:**
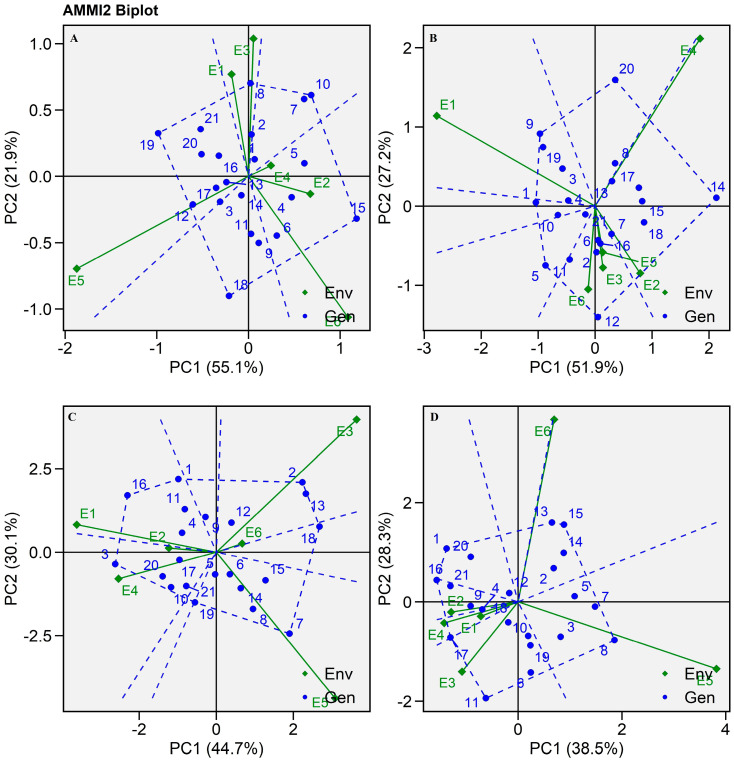
AMMI II biplot of 21 pearl millet hybrids (Blue text) evaluated in six environments (Green text) during summer-2021. Biplot developed from the values of PC I and PC II derived from AMMI ANOVA (**A**) Grain yield, (**B**) Dry fodder yield, (**C**) Iron content, (**D**) Zinc content.

In the current investigation, IPCA II was used to dissect a further G × E interaction because of its significant nature in the study (Table 2). The AMMI II biplot was created to investigate IPCA II (Figure 3). In this plot’s polygon view, the highest performance for a trait in a given environment was represented by the dotted line joining the vertex genotypes. The degree of interaction with the particular environment was shown by the vertical projection from the genotype to the environment vector. The biplot displayed E1 and E3 is highly interactive for SHT 108 (G8), which contributed largely to GEI for grain yield (Figure 3A). Similarly, E2, E4, and E6 are highly interactive environments for hybrids SHT 115 (G15), SHT105 (G5), and SHT 104 (G4). Among all the hybrids, SHT 104 produced exceptionally higher dry fodder (Table S5). Similarly for Fe content, SHT 109 and SHT 119 are highly responsive at E1 for dry fodder yield (Figure 3B). SHT 103 and SHT 113 were highly interactive towards variation in E3 and E6, respectively, while SHT116 at E1 and E3 and SHT 102 at E6 were the most favorable environments for the hybrids SHT 102 and SHT 113, but E6 was found to be the least interactive environment compared to E3 for Fe content (Figure 3C). Similarly, E5 was also found to be highly interactive towards GEI for hybrid SHT 107 (G7). Zn content was found to be highly interactive due to hybrid SHT 108 in environment E5, which has the longest vector indicating a highly variable or interactive environment for Zn content. Similarly, E6 was found to be interactive for hybrids SHT 113, SHT 114, and SHT 115, while other locations possessed short vectors with the least interaction indicating the nearly stable absorption of Zn from soils in particular locations (Figure 3D). In this way, Asungre et al. [16] described an AMMI II biplot and displayed high grain yield-producing hybrids ICMH 177017 and ICMH 177018 for the Manga 2018 environment and ICMH 177016 for the Denugu 2019 environment. They reported ICMH IS 16052 and ICMH IS 16187 as highly stable hybrids for Fe and Zn content, respectively, according to AMMI II biplot analysis.

### 2.4. Correlations among Traits over Environments

**Figure 4:**
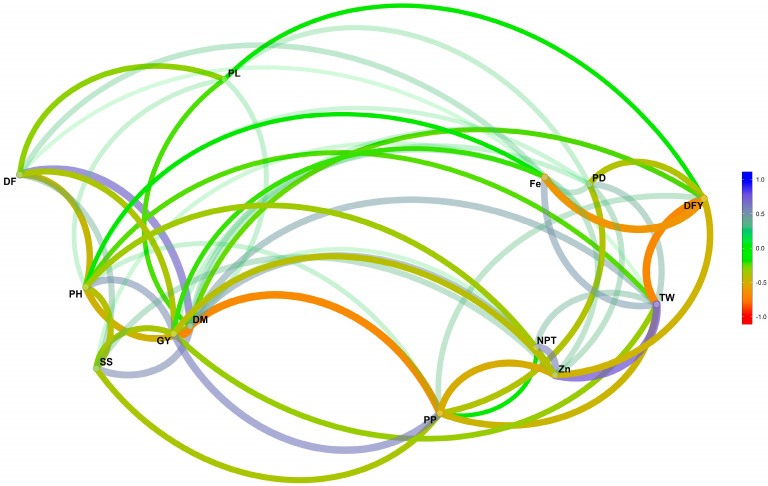
Pearson’s correlation network plot. DF: days to 50% flowering, DM: days to maturity, PH: plant Height, NPT: number of productive tillers per plant, PL: panicle length, PD: panicle diameter, TW:1000 seed weight, PP: plant population at harvest, SS: seed set percentage under bagging condition, GY: grain yield, DFY: dry fodder yield, Fe: iron content, Zn: zinc content.

Pearson’s correlation was performed, and a network plot of correlation was developed as per Singamsetti et al. [22] among all traits using mean over environment data (Table S6 and Figure 4). Figure 4 indicates the positive and significant correlation between DF and DM while GY is positively correlated with PH and PL while, it is negatively correlated with Fe and Zn content. The network plot indicated that Fe, PD, DFY, TW, NPT, Zn, and PP were nearby in one cluster. Similarly, GY, DM, SS, PH, and DF are nearby in another cluster. This proximity of the trait was determined using multidimensional clustering suggested by Gower [23]. A similar Pearson’s correlation was performed by Sanjana Reddy et al. [15] among yield, quality, and agronomic traits. They observed a negative correlation of Fe and Zn with grain yield (−73 and −91, respectively) and a strong correlation between Fe and Zn with a value of 0.82.

### 2.5. GGE Biplots

#### 2.5.1. Which-Won-Where and What?

**Figure 5:**
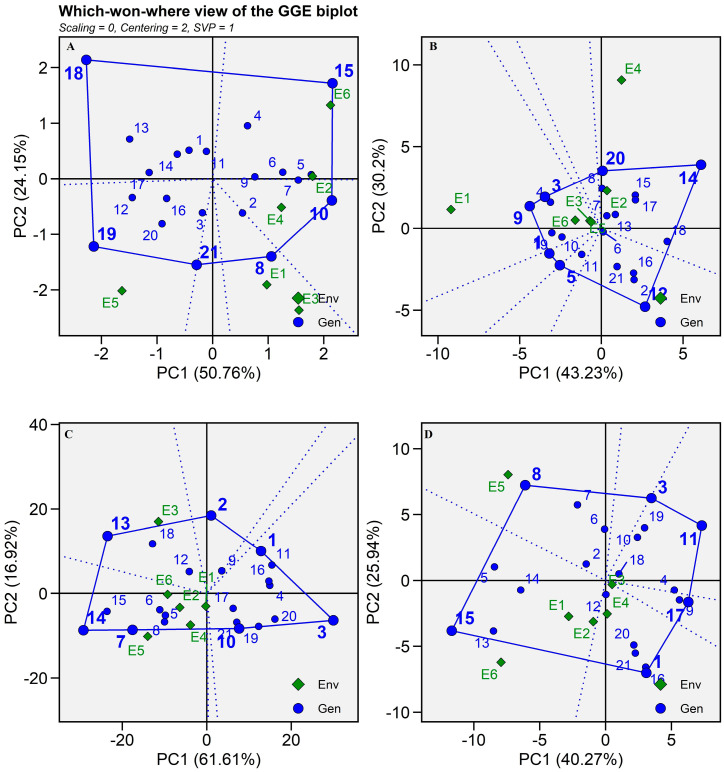
Which-won-where view of 21 pearl millet hybrids (Blue text) evaluated in six environments (Green text) during summer-2021. (**A**) Grain yield, (**B**) Dry fodder yield, (**C**) Iron content, (**D**) Zinc content.

The best hybrid for a specific location can be predicted by the “Which-won-where and what?” biplot. In this biplot, the polygon is developed by joining the vertex hybrids (Figure 5) [24]. The rays (dotted lines) start at the plot’s origin and go perpendicular to the polygon’s sides, further splitting the polygon into many distinct sectors. It was suggested by Gauch and Zobel [25] that this distinction supports the recommendation of a genotype for a certain environment. According to Memon et al. [9], the genotype at the vertex is the one that performs best in that sector of the environment. The biplot depicted four mega environments for grain yield. E3 and E1 come under a common mega environment in which SHT 108 performed better than other environments for grain yield (Table S4). Similarly, E2 and E4 construct a common mega environment in which SHT 110 is recommended as a winning hybrid (Figure 5A and Table S4).

E6 and E5 solely construct their separate mega environment with SHT 115 and SHT 119 as a winning hybrid, respectively. Hybrids SHT 111, SHT 109, and SHT 101 fall near the origin of the biplot and showed the least interaction with environments while SHT 118 did not fall under any mega environment, indicating lower grain yield-producing hybrid in most of the environments (Figure 5A). There were two mega environments found for dry fodder yield (Figure 5B). E2 and E4 environments come under a single mega environment with SHT 114 and SHT 120 as competitive hybrids. Similarly, the rest of the environments created a single mega environment with SHT 103 and SHT 109 as winning hybrids. However, hybrids SHT 101, SHT 105, and SHT 112 did not participate in any mega environment indicating lower performance across environments. As per Figure 5C, E2, E4, E5, E6, and E1 form a single mega environment for SHT 114 and SHT 107 for Fe content. E3 solely created a single mega environment for SHT 113 for Fe content (Figure 5C). Hybrid SHT 108 falls under the single mega environment E5, while SHT 115 was highly interactive in E6, E1, and E2 environments as these environments fall under the single mega environment for Zn content (Figure 5D). In this biplot, E3 fall at the origin indicated that all hybrids did not interact with this environment for Zn content (Figure 5D).

This outcome was matched by Sanjana Reddy et al. [15] in pearl millet by creating a single mega environment of Mandor, Durgapura, Coimbatore, Aurangabad, Ludhiana, and Ananthapuram for vertex pearl millet varieties MP595 and MP 596 for grain yield. Moreover, they observed that most of the locations clustered over the origin of the biplot for dry fodder yield except New Delhi, Gurugram, Ludhiana, Niphad, and Perumallapalle locations in India.

#### 2.5.2. Mean Seed Yield vs. Stability

**Figure 6:**
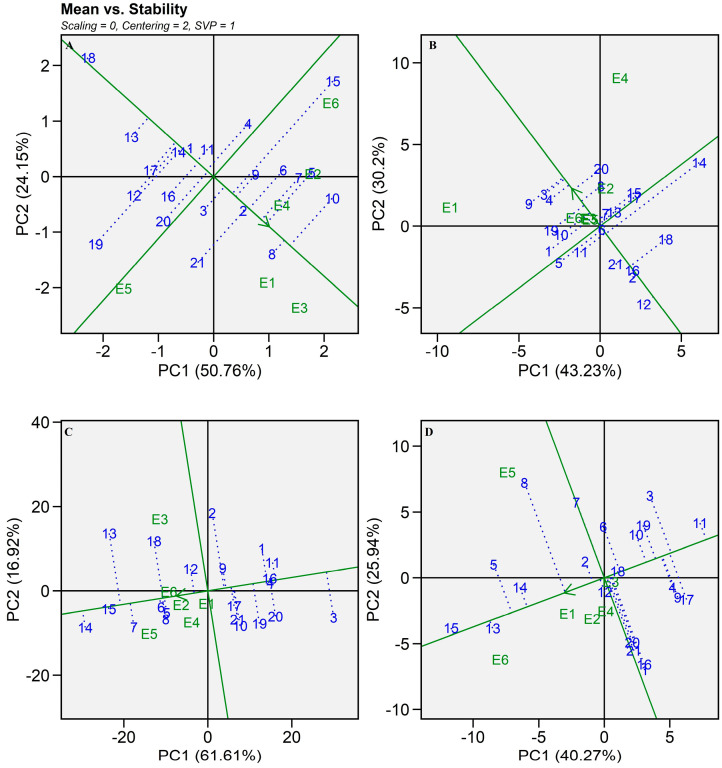
Average environment coordination (AEC) view of the GGE-biplot based on environment-focused scaling and genotype-focused singular-value partitioning for the mean performance and stability of 21 pearl millet hybrids (Blue text) evaluated in six environments (Green text) during summer 2021. (**A**) Grain yield, (**B**) Dry fodder yield, (**C**) Iron content, (**D**) Zinc content.

A two-dimensional plot comparing grain yield against stability was created based on PC I and PC II scores as per Patel et al. [26] and Memon et al. [9]. It is perfect for genotype evaluation because the row metric preservation technique was used to construct this plot (Figure 6). Using an average environment coordination (AEC) perspective, the stability of the genotypes was displayed. An arrow on the AEC line indicated the direction of the increasing mean. Very stable genotypes were indicated by a short line drawn perpendicular to the AEC axis. As per Figure 6A, hybrid SHT 108 was stable and good in grain yield production while SHT 110 was found to be a high-performing hybrid across all the environments, but it showed an interaction with environmental factors (Figure 6A). Likewise, SHT 103, SHT 104, and SHT 109 were better-performing hybrids with good stability for dry fodder yield (Figure 6B). SHT 115 was placed on the AEC line with the shortest vector in a biplot for Fe and Zn content, which indicated perfect stability with a higher micronutrient content as it fell on the AEC line in the increasing direction (Figure 6C,D). Sanjana Reddy et al. [15] used this biplot (mean seed yield versus stability plot) to decipher high grain yield-producing pearl millet variety MP596 near the ideal location of Ananthapuram. Similarly, a comprehensive comparison between Eberhart and Russell’s [27] joint regression and GGE biplot analyses was carried out by Alwala et al. [28] to identify stable and high-yielding maize hybrids.

#### 2.5.3. Discriminativeness vs. Representativeness

**Figure 7:**
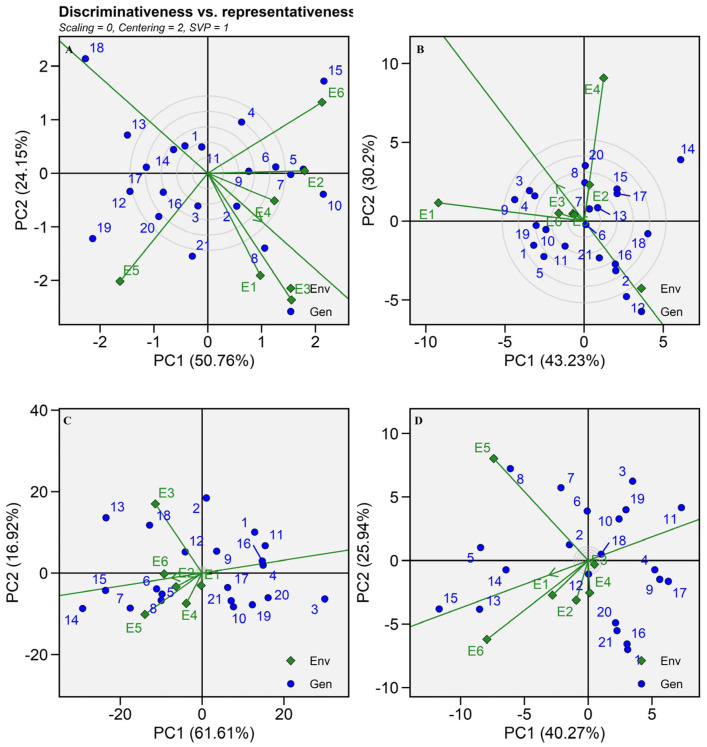
GGE biplot view of the discriminativeness and representativeness developed using environment-focused centering and symmetrical method of singular-value partitioning of 21 pearl millet hybrids (Blue text) evaluated in six environments (Green text) during summer 2021. (**A**) Grain yield, (**B**) Dry fodder yield, (**C**) Iron content, (**D**) Zinc content.

The cosine angle between two environmental vectors explains the link between them. According to Memon et al. [9], an acute angle denotes a positive relationship while an obtuse angle denotes a negative correlation, and a perfect 90° angle shows no correlation between environments. Patel et al. [26] stated that the discrimination capacity of an environment is shown by the length of its vector on the biplot, which is proportionate to the standard deviation of the same environment. To evaluate the representativeness of the environment, one must apply the average environment axis (AEA). As the angle between the environmental vector and the AEA increases, representativeness reduces and vice versa. In this study, E6, E4, and E2 were arranged with an acute angle between them, which showed a positive correlation among them for grain yield production but showed no correlation between E3 and E1 as they created a nearly right angle between them. E5 was negatively correlated as it made a great obtuse angle with E6 for grain yield per plant (Figure 7A). Similarly, these two environments were referred to as non-representative environments because they were plotted at a nearly right angle to AEA (Figure 7A). As per Figure 7B, E1 and E4 were highly interactive individually with dry fodder yield but they had no relation as per the above discussion. E3 and E5 were found to be interactive environments for Fe content while other environments were non-interactive and stable in grain Fe content accumulation (Figure 7C). Likewise, E5 and E6 were highly interactive for Zn content but no relationship was established between them due to the right angle (Figure 7D). This biplot also suggests the idea of ideal hybrids from the circles around the origin [10]. Generally, the best and ideal genotypes fall inside the innermost circle. As per the biplot, SHT 111 was ideal for grain yield production while SHT 107 and SHT 106 were ideal for dry fodder yield. There were no ideal hybrids for Fe and Zn content as they created a very small circle around the origin (Figure 7C,D).

#### 2.5.4. Y × WAAS Biplot

**Figure 8:**
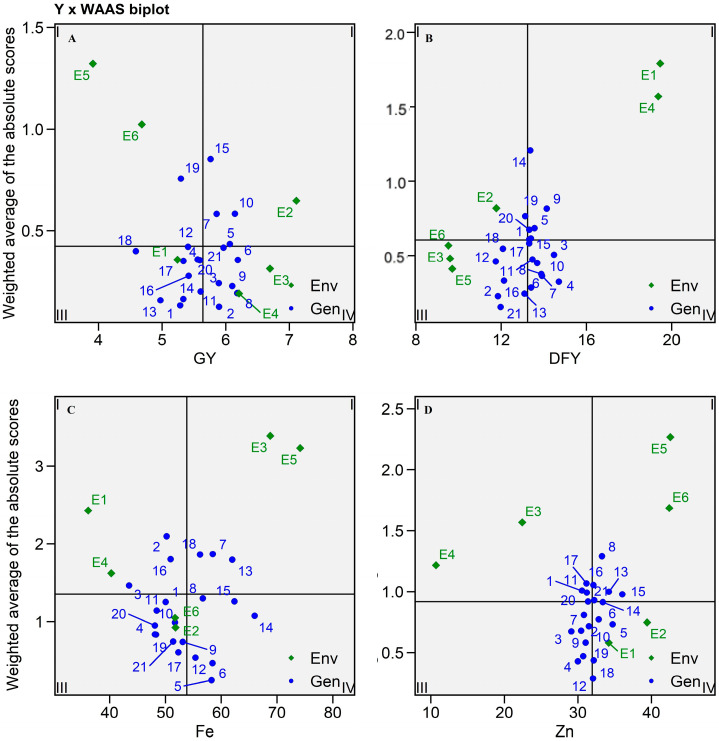
Mean performance × WAASB biplot based on combined interpretation of productivity and stability (WAASB) for 21 pearl millet hybrids (Blue text) evaluated in six environments (Green text) during summer-2021. (**A**) Grain yield, (**B**) Dry fodder yield, (**C**) Iron content, (**D**) Zinc content.

The Y × WAAS plot separates hybrids into four quadrants (Figure 8, Table 3 and Table S5), sanctioning for concurrent hybrid selection with higher values of trait and stability of the included variables [26,29]. According to the biplot (Figure 8A), SHT 119 and SHT 112 including E5 and E6 fell in the first quadrant and verified more instability of hybrids with little productivity and a robust discriminating ability for grain yield, whereas SHT 115, SHT 110, SHT 107, and SHT 105 including E2 were in the second quadrant and presented meager stability and higher GY, which guided additional attention to this environment for increasing grain yield as per Patel et al. [26] (Figure 8A). The third quadrant included nine hybrids with the E1 environment, which exhibited higher stability (indicated by a low value of WAAS) but comparatively lower productivity. In contrast, the remaining eight hybrids located in the fourth quadrant displayed both greater stability and exceptional performance in grain yield production. Similarly, for dry fodder yield, three hybrids including E1 and E4 were found in quarter I, four hybrids including E2 were in quarter II, and seven hybrids each were in quarters III and IV, with the remaining environments in quarter III (Figure 8B). All 21 hybrids were also divided into four quarters for Fe and Zn content (Figure 8C,D). For Fe content, three hybrids each were allocated to quarters I and II while nine and six hybrids were allocated to quarters III and IV, respectively (Figure 8C). Similarly, five hybrids each were allocated to quarters I, II, and IV, and the remaining seven hybrids were allocated to quarter III for Zn content (Figure 8D).

### 2.6. Multi-Trait Stability Index (MTSI)

Memon et al. [9] stated that conventional stability parameters like the Eberheart and Russel model [27] have been applied by mainstream plant breeders. It might not be possible to offer a straightforward explanation of mean performance and trait stability with the selection of a stable genotype based just on mean, regression, and deviation from regression parameters. Because of this limitation, the multi-trait stability index method has developed into an advanced quantitative genetics tool that can be used to find and use favorable variants in any type of crop using multiple traits [12].

**Figure 9:**
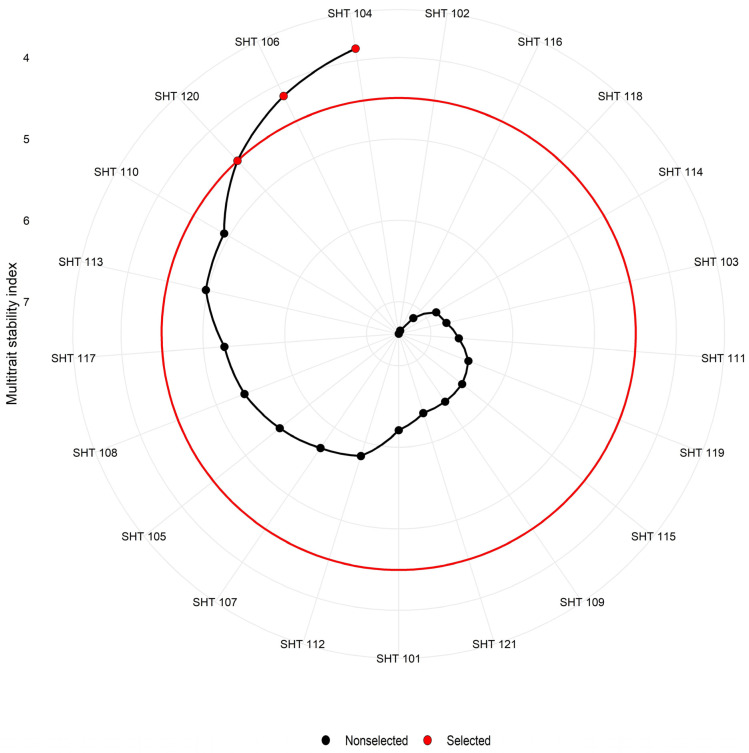
Hybrid ranking and selected hybrids among 21 pearl millet hybrids for multi-trait stability index (MTSI) view considering 15% selection intensity.

In this study, all traits included in the MTSI calculation were highly significant for GEI in the joint ANOVA except plant population at harvest and the number of productive tillers per plant [22]. Pearson’s correlation matrix was used to generate the WAASBY (Weighted Average of Absolute Scores of Stability with Yield) value and retrieve a high-magnitude relationship that was combined as a common factor. In this study, data from eleven characters were used to perform exploratory factor analysis. This analysis generated four PCs with 76.69% cumulative variance (Table 4). Communality reflected shared variance among traits ranging from 0.536 (PL) to 0.933 (DM) with a mean value of 0.767 after varimax rotation. Specific variance relates to the part of the variability not shared with other variables due to a unique factor that was found higher for PL and lower for DM (Table 4). The traits included in the analysis were categorized into four factors by extracting the WAASBY value from each corresponding character (Table 5). DF, DM, and DFY were clustered in FA1; PL, SS%, and Fe were in FA2; TSW and GY were in FA3; and PH, PD, and Zn were in FA4 (Table 5). Selection criteria for mean performance was set as the nature of traits like DF and DM were negatively selected and the rest of the characters were selected in a positive direction. Similarly, the WAASBY value was extracted by giving equal weight to the mean and stability. The selection differential for mean performance for DF and DM showed a gain in the negative direction leading to the selection of early-maturing hybrids. Likewise, DFY, GY, PH, TSW, and SS% showed positive selection differentials indicating the selection of hybrids in increasing grain yield and yield attributing traits, except for PD because it was negatively correlated with PL for selected hybrids (Table 4). From this selection, stability and mean performance (WAASBY) were positively selected exclusively for PL. The selection conducted in Figure 9 serves as the foundation for calculating the mean of the chosen genotypes (Xs), which generally aligned with expectations. However, an exception was observed for Fe and Zn, as they exhibited a negative correlation with grain yield. A comparable approach was employed to assess the relative impacts of moisture stress in maize by Singamsetti et al. [30] and Koundinya et al. [31], who used MTSI to evaluate genotype–environment interactions, including leaf area index, yield per plant, harvest index, dry matter, and starch yield per plant in 25 cassava genotypes.

The selection of stable hybrids with higher mean performance for multiple variables is the most important part of stability analysis [32]. Genotype–ideotype Euclidian distance-based scores were used to carry out exploratory factor analysis. Exploratory factor analysis and ideotype estimates yielded scores for 21 hybrids in the first four factors (Table S7).

MTSI aids in the selection of genotypes by identifying those with both higher stability and superior mean performance across all significant interacting variables. Also, Memon et al. [9] identified castor accession using MTSI with desired values for six yield and attributing and biochemical traits. Hybrids with lower MTSI values are chosen in the selection process, with a selection intensity of 15%. In the current investigation, SHT 104 with an MTSI of 3.853, SHT 106 with an MTSI of 4.153, and SHT 110 with an MTSI of 4.931 were chosen due to their maximum stability and high mean performance across the analyzed traits (Figure 9 and Table S7). In Figure 9, the red circle signifies the cutoff point with an MTSI value of 4.931, while SHT 102 exhibited a higher MTSI value of 7.393, followed by SHT 116 (MTSI = 7.351), SHT 118 (MTSI = 7.129), and SHT 114 (MTSI = 6.86) (Table S7). These hybrids were identified as unstable, demonstrating poor performance for the traits under current investigation. Likewise, Koundinya et al. [31] chose a set of highly stable twenty-five cassava genotypes to enhance the performance of key parameters, including leaf area index, yield per plant, harvest index, dry matter, and starch yield per plant. Yadav et al. [33] adopted a WAASBY-based MTSI approach and selected ICMH-177111 and CMX-207137 genotypes across five environments for 54 health-benefiting metabolites. A similar approach was adopted to evaluate the relative effects of drought and saline stress on seed germination in sweet sorghum [34] and cowpea [35].

MTSI helps to select hybrids with a greater mean for various traits with considerable stability [36]. It selected SHT 120, SHT 106, and SHT 104 as higher performers for most of the traits. Among them, SHT 106 was spotted in the fourth quadrant of the Y × WAAS biplot for GY, dry fodder yield, and mineral content, indicating great performance with better stability. Similarly, SHT 106 was the most ideal hybrid for dry fodder yield and grain yield. SHT 120 was found to be a moderately stable and higher performer for dry fodder yield, while SHT 108 was ideal for grain yield production. Likewise, SHT 115 was superior in grain Fe and Zn concentration across all locations under study as per the Mean vs. Stability biplot. The Which-Won-Where and What biplot for grain yield identified SHT 106 as a higher performer for the mega environment of E2, E4, and E6. while it was found to be a stable performer for dry fodder yield for all environments. It was also moderately interactive for grain Fe and Zn content. A parallel approach to genotype selection was employed by Memon et al. [9] in their study on castor. The selected genotypes can contribute to a more stable genetic basis for future breeding programs for large tracts.

## 3. Materials and Methods

### 3.1. Field Trials

The field experiment for the evaluation of 21 pearl millet hybrids (Table S1) was conducted during summer 2021 across six locations in India: E1 Mandor, Rajasthan (26.3427° N, 73.0443° E), E2 Jamnagar, Gujarat (22.4707° N, 70.0577° E), E3 Sardarkrushinagar, Gujarat (24.3260° N, 72.3169° E), E4 Ahmedabad, Gujarat (23.0225° N, 72.5714° E), E5 National Agricultural Research Project (NARP), Aurangabad, Maharashtra (19.8556° N, 75.3015° E), and E6 Coimbatore, Tamilnadu (11.0168° N, 76.9558° E). Among them, E1, E2, E3, E4, and E5 fall under the western plain and Hills agro-climatic zone while E6 falls under the Eastern Coastal Plains and Ghats agro-climatic zone of India as per the Indian Council of Agricultural Research, India.

### 3.2. Experimental Design and Package of Practices

The experiment employed a Randomized Complete Block Design (RCBD) with three replications at each location. Each genotype was raised in six rows, each measuring 4 m in length. The between-row spacing was 60 cm at E1 and E2, 45 cm at E3, and 50 cm at E4, E5, and E6. To mitigate potential damage and border effects, data collection was conducted by excluding the border rows from each plot. The experiment was performed adhering to essential agronomic practices and plant protection methods. To manage initial weed concerns, Pendimethalin 30% EC was applied as a pre-emergence herbicide shortly after planting. Experimental details like sowing date, spacing, and plot size are given in Table S2.

### 3.3. Trait Phenotyping

Phenotyping for the days to 50% flowering (DF) and days to maturity (DM) was conducted on a plot basis. Five plants were randomly selected, and plant height (PH), panicle length (PL), and panicle diameter (PD) were measured in centimeters, grain yield (GY) and dry fodder yield (DFY) were measured in kilograms per net plot, 1000-seed weight (TSW) was measured in grams, and productive tillers per plant (PTPP) were counted as per Sanjana Reddy et al. [15]. Iron (Fe) and Zinc (Zn) content in grains were measured in mg per kg as per Kumar et al. [1]. Plant population at harvest is a clear indicator of effective plants that contribute to the final yield, and it is important for the evaluation of hybrids to determine the survival rate of the hybrid population. This was measured by counting productive plants per net plot at harvesting time. The seed set under bagging conditions was measured as a percentage, which indicated the hybrid sterility at the field level and directly correlated to grain yield per plant. Pearl millet is a cross-pollinated crop, but this measurement indicated the fertility restoration capacity of the male parent that was used to develop the hybrid.

### 3.4. Analysis of Variance

The data on seed yield and related traits across six test environments were subjected to a joint analysis of variance. A mixed-effect model was employed, with hybrids considered fixed factors and environments treated as random factors, following the methodology outlined by Pratibha et al. [29].

### 3.5. GEI Analysis

#### 3.5.1. AMMI Analysis

The yield data (GY) were analyzed using the AMMI method, following Bradu and Gabriel [37]. A basic analysis of variance provides insights into the additive main effects of hybrids and locations. Conversely, the principal component analysis (PCA) unveils the non-additive components within the experimental framework. The significance of the location-wise stable hybrids identified through AMMI analysis was assessed using the F-test. AMMI biplots were generated using the primary impact of means against the principal component (PC) axis I and between the first two PC axes. According to Farshadfar [38] and Atta et al. [39], hybrids were also ranked using AMMI stability values (ASV) and the yield stability index (YSI).

#### 3.5.2. GGE Biplots

Yan [40] and Zobel et al. [41] asserted that the site regression genotype–genotype–environment interaction (GGEI) biplot models serve as a robust means for effectively analyzing and interpreting the complex data structures in multi-environment scenarios in crop improvement. In the GGE study, it was found that the first two components are the optimal choices for constructing GGE biplots as they capture the majority of the variance [42]. Every biplot in the study was generated using environment-centered data and the symmetrical singular value partitioning (SVP) approach. A biplot of the mean versus stability was created using the row metric preservation method of SVP.

### 3.6. Multi-Trait Stability Index (MTSI)

To evaluate the stability of each hybrid, a Singular Value Decomposition (SVD) of the matrix of Basic Linear Unbiased Predictions (BLUPs) for the GEI effects was generated using a linear mixed model (LMM). The stability of each hybrid was ascertained by computing the Weighted Average of Absolute Scores (WAAS) using the Singular Value Decomposition (SVD) of the matrix of BLUPs for the GEI effects. This was obtained using a linear mixed-effect model. Simultaneous selection for both mean performance and stability was achieved by employing the Weighted Average of Absolute Scores by Yield (WAASBY) index [12].

The hybrid with the lowest MTSI score demonstrates a closer resemblance to the ideotype, indicating superior mean performance and stability across various environments for all studied traits. The selection of the most desirable hybrids, characterized by optimal production and stability, was carried out with a selection intensity of 15% using the following equation:

$$MTSI_{i}=\overset{f}{\underset{j=1}{\sum }}{\left[{\left(F_{ij}-F_{j}\right)}^{2}\right]}^{0.5}$$

where, *MTSI_i_* is the multi-trait stability index for the *i*th genotype, *F_ij_* is the *j*^th^ score of the *i*th genotype, and *F_j_* is the *j*th score of the ideotype. A plot of *MTSI* scores was generated to illustrate the differentiation between the selected and non-selected genotypes.

The A Y × WAAS biplot was created to categorize hybrids into four distinct groups, facilitating a comprehensive interpretation of both stability and mean performance across diverse contexts. This four-quadrant biplot was constructed with grain yield on the *x*-axis and WAASB values on the *y*-axis.

### 3.7. Statistical Packages

The pooled analysis of variance, AMMI analysis, GGE biplots, and MTSI computations were conducted using RStudio, specifically with R version 4.0 [43]. The “metan” R package was utilized for these analyses [44].

## 4. Conclusions

Biological yield in pearl millet is highly influenced by environmental factors or a changing environment. In the current study, the evaluated material showed considerable variability across all the locations for most of the characteristics. These hybrids showed the significant contribution of GEI for most of the characteristics except the number of productive tillers per plant and plant population at harvest. Pearson’s correlation indicated the positive and significant correlation among agronomic characters and the negative correlation with the grain Fe and Zn content. AMMI and GGE biplot analysis helped to select the dual-purpose hybrid SHT 106 and the SHT 115 hybrid for superior grain Fe and Zn concentration. Similarly, SHT 108 was selected for higher and more stable grain yield. E2, E3, E5, and E6 were identified as the least interactive environments for dry fodder yield and highly interactive for grain yield per plant, which indicated that the stable performance of the fodder production affects the grain production. On the basis of the discriminativeness and representativeness biplot, SHT 111 and SHT 106 were selected as ideal hybrids for grain yield production and dry fodder yield production, respectively. across all evaluated locations, while SHT 115 was found to be highly stable and have high Fe and Zn across most of the locations as per the mean vs. stability analysis. Similarly, SHT 120, SHT 106, and SHT 104 were selected through MTSI as superior hybrids for stability and for most of the agronomical traits across all evaluated locations. This selection was also supported by selecting SHT 106 as a stable and high-mean-performing hybrid for GY, dry fodder yield, and mineral content using the Y × WAAS biplot.

## Figures and Tables

**Table 1:** Combined analysis of variance for yield and its attributing traits towards total variation among 21 pearl millet hybrids tested in six environments in summer-2021.

Source of Variation	Environment (E) (df: 5)		Genotype (G) (df: 20)		GEI (df: 100)		Residual (df: 240)	CV (%)	Mean over Environments
Trait	Mean Squares	% (G + E + GEI)	Mean Squares	% (G + E + GEI)	Mean Squares	% (G + E + GEI)	Mean Squares		
DF	1719.09 **	74.88	78.24 **	13.63	13.18 **	11.48	1.96	2.52	55.49
DM	3062.49 **	86.56	58.86 **	6.66	11.99 **	6.78	1.62	1.46	87.42
PH	27,729.09 **	69.32	1656.50 **	16.56	282.34 **	14.12	48.01	3.59	193.04
PTPP	27.35 **	80.31	0.47 **	5.6	0.24	14.09	0.21	21.48	2.17
PL	87.49 **	19.47	54.80 **	48.79	7.12 **	31.73	1.20	4.06	26.92
PD	1.92 **	32.65	0.57 **	38.8	0.08 **	28.54	0.02	4.45	3.27
TSW	348.59 **	86.64	6.93 **	6.89	1.30 **	6.47	0.22	4.84	9.71
PP	121,726.20 **	96.04	264.28	0.83	198.29	3.13	173.38	7.18	183.26
SS%	12,733.15 **	49.54	1638.85 **	25.5	320.76 **	24.96	85.02	12.22	75.36
GY	96.51 **	69.19	3.27 **	9.39	1.49 **	21.42	0.34	10.40	5.64
DFY	1486.61 **	87.51	12.79 **	3.01	8.05 **	9.48	1.14	8.07	13.24
Fe	14,388.46 **	74.38	582.83 **	12.05	131.30 **	13.57	48.87	12.99	53.80
Zn	10,402.73 **	92.41	50.18 **	1.78	32.67 **	5.81	15.22	12.21	31.93

** Significant at *p* ≤ 0.01, respectively, df: degrees of freedom, GEI: genotype–environment interaction, CV% coefficient of variation, DF: days to 50% flowering, DM: days to maturity, PH: plant height, PTPP: Productive tillers per plant, PL: panicle length, PD, panicle diameter, TSW: 1000 seed weight, PP: plant population at harvest, SS%: seed set under bagging condition, GY: grain yield, DFY: dry fodder yield, Fe: grain iron content, Zn: grain zinc content.

**Table 2:** Principal components and their contributions as per AMMI model for G × E interactive traits across six locations (Summer 2021).

Source of Variation	PC1 (df: 24)		PC2 (df: 22)		PC3 (df: 20)		PC4 (df: 18)		PC5 (df: 16)	
Trait	Mean Square	Contribution (%)	Mean Square	Contribution (%)	Mean Square	Contribution (%)	Mean Square	Contribution (%)	Mean Square	Contribution (%)
DF	28.49 **	51.90	15.84 **	26.40	8.53 **	13.00	3.92 *	5.40	2.76	3.40
DM	23.25 **	46.50	14.92 **	27.40	7.91 **	13.20	5.33 **	8.00	3.67 **	4.90
PH	482.53 **	41.00	314.55 **	24.50	294.52 **	20.90	128.43 **	8.20	95.69 *	5.40
PL	9.83 **	33.10	9.32 **	28.80	7.40 **	20.80	4.85 **	12.30	2.25 *	5.10
PD	0.13 **	39.00	0.10 **	28.30	0.07 **	17.90	0.04 **	9.60	0.02	5.20
TSW	2.43 **	45.00	1.66 **	28.20	0.82 **	12.70	0.68 **	9.40	0.38 *	4.70
SS%	694.38 **	52.00	316.93 **	21.70	258.18 **	16.10	135.73	7.60	51.99	2.60
GY	3.43 **	55.10	1.48 **	21.90	1.01 **	13.60	0.52	6.30	0.29	3.10
DFY	17.42 **	51.90	9.96 **	27.20	3.50 **	8.70	3.18 **	7.10	2.53 **	5.00
Fe	244.57 **	44.70	179.59 **	30.10	97.33 **	14.80	52.27	7.20	26.34	3.20
Zn	52.37 **	38.50	42.05 **	28.30	28.68 *	17.60	24.24	13.40	4.71	2.30

*, ** Significant at *p* ≤ 0.05 and *p* ≤ 0.01, respectively, df: degrees of freedom, PC: principal component, DF: days to 50% flowering, DM: days to maturity, PH: plant height, PL: panicle length, PD: panicle diameter, TSW: test weight, SS%: seed set under bagging condition, GY: grain yield, DFY: dry fodder yield, Fe: grain iron content, Zn: grain zinc content.

**Table 3:** PC value, mean over environment, and WAAS score of 21 hybrids evaluated across six different locations during summer-2021.

Hybrid	Mean over Environment	PC1	PC2	PC3	PC4	PC5	WAAS
SHT 101	5.285	0.068	0.128	−0.394	−0.496	−0.562	0.132
SHT 102	5.897	0.034	0.315	0.187	−0.028	0.084	0.125
SHT 103	5.893	−0.309	−0.192	0.049	0.336	−0.352	0.242
SHT 104	5.559	0.472	−0.158	−0.217	−0.339	−0.089	0.358
SHT 105	6.065	0.611	0.099	−0.259	0.404	0.153	0.434
SHT 106	6.192	0.310	−0.447	0.393	0.144	−0.071	0.356
SHT 107	5.859	0.609	0.582	0.483	−0.072	−0.039	0.583
SHT 108	6.182	0.022	0.702	0.068	−0.290	0.267	0.193
SHT 109	6.103	0.113	−0.501	−0.251	−0.263	0.258	0.228
SHT 110	6.146	0.685	0.611	0.128	0.101	0.016	0.584
SHT 111	5.606	0.029	−0.433	−0.522	0.060	0.006	0.201
SHT 112	5.408	−0.605	−0.211	−0.009	0.377	0.069	0.421
SHT 113	4.974	−0.239	−0.045	−0.005	−0.491	−0.164	0.157
SHT 114	5.335	−0.077	−0.143	0.551	0.407	0.118	0.164
SHT 115	5.764	1.178	−0.320	−0.388	0.130	0.191	0.852
SHT 116	5.419	−0.322	0.155	−0.298	0.419	−0.229	0.278
SHT 117	5.337	−0.352	−0.087	0.771	−0.300	0.127	0.351
SHT 118	4.589	−0.211	−0.901	0.353	−0.236	0.075	0.399
SHT 119	5.296	−0.984	0.324	−0.527	−0.115	0.600	0.756
SHT 120	5.595	−0.512	0.166	−0.021	0.058	−0.231	0.355
SHT 121	5.966	−0.520	0.355	−0.092	0.191	−0.230	0.416
E1	5.242	−0.185	0.769	−0.393	−0.625	0.677	0.357
E2	7.110	0.675	−0.132	1.365	0.003	0.175	0.647
E3	6.695	0.055	1.038	−0.195	0.895	−0.221	0.313
E4	6.208	0.245	0.082	−0.150	−0.695	−0.819	0.192
E5	3.912	−1.874	−0.696	0.090	0.153	−0.006	1.321
E6	4.681	1.084	−1.061	−0.717	0.269	0.193	1.023

PC: principal component, WAAS: weighted average of absolute score, E1: Mandor, E2: Jamnagar, E3: S. K. Nagar, E4: Ahmedabad, E5: NARP, Aurangabad, E6: Coimbatore.

**Table 4:** Eigenvalues, explained variance, factorial loadings after varimax rotation, communalities, and uniqueness obtained in the factor analysis of the six GEI significant variables studied in 21 pearl millet hybrids across six environments during summer 2021.

Traits	FA1	FA2	FA3	FA4	Communality	Uniqueness
DF	−0.888	−0.007	0.152	0.039	0.813	0.187
DM	−0.952	0.164	−0.001	0.007	0.933	0.067
PH	−0.401	−0.481	0.001	−0.563	0.709	0.291
PL	−0.229	−0.682	0.131	−0.033	0.536	0.464
PD	0.099	−0.011	−0.098	−0.840	0.725	0.275
TSW	−0.154	−0.043	−0.903	−0.130	0.857	0.143
SS%	0.161	−0.749	0.155	−0.331	0.721	0.279
GY	−0.385	−0.283	0.584	−0.156	0.593	0.407
DFY	−0.721	−0.286	−0.099	−0.440	0.804	0.196
Fe	0.369	−0.716	−0.398	0.216	0.855	0.145
Zn	0.300	0.114	−0.544	0.701	0.891	0.109
Eigen value	3.61	2.16	1.53	1.13	0.767 ^a^	
Variance (%)	32.81	19.63	13.92	10.32		
Accumulated (%)	32.81	52.45	66.37	76.69		

^a^ Average of the communality. FA: factor Analysis, DF: days to 50% flowering, DM: days to maturity, PH: plant height, PL: panicle length, PD, panicle diameter, TSW: 1000 seed weight, SS%: seed set under bagging condition, GY: grain yield, DFY: dry fodder yield, Fe: grain iron content, Zn: grain zinc content.

**Table 5:** Selection differential for mean of the traits and WAASBY index for 11 traits of 21 pearl millet hybrids across six environments during summer 2021.

Traits	Factor	Mean Performance			WAASBY		
		Overall (Xo)	Selected Genotype (X_s_)	SD (%)	Overall (X_0_)	Selected Genotype (X_s_)	SD (%)
DF	FA 1	55.492	54.333	−1.159	0.498	0.297	−0.202
DM	FA 1	87.423	86.444	−0.979	0.532	0.295	−0.237
DFY	FA 1	13.239	13.812	0.573	0.495	0.436	−0.059
PL	FA 2	26.96	27.556	0.595	0.458	0.489	0.03
SS%	FA 2	75.399	76.852	1.452	1.099	1.054	−0.045
Fe	FA 2	53.82	51.556	−2.265	0.882	0.581	−0.301
TSW	FA 3	9.706	10.346	0.64	0.304	0.169	−0.134
GY	FA 3	5.641	5.782	0.14	0.312	0.298	−0.014
PH	FA 4	193.042	195.648	2.606	1.073	0.461	−0.612
PD	FA 4	3.269	3.183	−0.086	0.138	0.108	−0.030
Zn	FA 4	31.955	31.407	−0.548	0.559	0.463	−0.097

FA: Factor Analysis, DF: days to 50% flowering, DM: days to maturity, DFY: dry fodder yield, PL: panicle length, SS%: seed set under bagging condition, Fe: grain iron content, TSW: 1000 seed weight, GY: grain yield, PH: plant height, PD: panicle diameter, Zn: grain zinc content, SD: Selection differential and WAASBY: Weighted average of absolute scores of stability with yield. Xo: mean of all hybrids, Xs: mean of selected hybrids.

## Data Availability

Data are contained within the article and Supplementary Materials.

## Supplementary Materials

The following supporting information can be downloaded at: https://www.mdpi.com/article/10.3390/plants13081101/s1, Table S1: List of hybrids used in the study with its description. Table S2: Experimental details. Table S3: Mean and range of quantitative traits of pearl millet hybrid in six environments in summer 2021 DF: days to 50% flowering, DM: days to maturity, PH: plant height, PTPP: number of productive tillers per plant, PL: panicle length, PD, panicle diameter, TSW: 1000 seed weight, PP: plant population at harvest, SS%: seed set under bagging condition, GY: grain yield, DFY: dry fodder yield, Fe: grain iron content, Zn: grain zinc content. Table S4: location-wise mean performance of all 21 pearl millet hybrids in summer 2021 DF: days to 50% flowering, DM: days to maturity, PH: plant height, PTPP: number of productive tillers per plant, PL: panicle length, PD, panicle diameter, TSW: 1000 seed weight, PP: plant population at harvest, SS%: seed set under bagging condition, GY: grain yield, DFY: dry fodder yield, Fe: grain iron content, Zn: grain zinc content. Table S5: PC value, mean across environments, and WAAS score for dry fodder yield, Fe content and Zn content of 21 hybrids evaluated across six different locations PC: principal component, WAAS: weighted average of absolute score, E1: Mandor, E2: Jamnagar, E3: S. K. Nagar, E4: Ahmedabad, E5: NARP, Aurangabad, E6: Coimbatore. Table S6: Pearson’s correlation coefficient among all traits across environments DF: days to 50% flowering, DM: days to maturity, PH: plant height, NPT: number of productive tillers per plant, PL: panicle length, PD: panicle diameter, TSW: 1000 seed weight, PP: plant population at harvest, SS: seed set under bagging condition, GY: grain yield, DFY: dry fodder yield, Fe: grain iron content and Zn: grain zinc content. Table S7: Genotype–ideotype (ID) scores, MTSI values for the 21 pearl millet hybrids for the first four factors, and relative contribution of each factor towards the MTSI FA: factor analysis, RC: relative contribution and MTSI: multi trait stability index.
