# Supplementary material for: Stability Analysis and Identification of Superior Hybrids in Pearl Millet [Pennisetum glaucum (L.) R. Br.] Using the Multi Trait Stability Index
Source: Plants (Basel). 2024 Apr 15;13(8):1101. doi: 10.3390/plants13081101 (PMC11053410; doi:10.3390/plants13081101)
Supplement: Supplementary file 1 [file plants-13-01101-s001.zip › plants-2894376-supplementary.pdf]

Table S1: List of hybrids used in the study with its description

S.No.	Test Code	Project Code	Identity	Pedigree	Institution
**1**	SHT 101	MSH 361	SVPMH-101	SVMSA-112 x SVR-50	Shakti Vardhak Hyb. Seed Pvt. Ltd.
**2**	SHT 102	MSH 363	BLPMH 109	BLPMA002 x BLPMR1414	Bisco BioSciences Pvt. Ltd.
**3**	SHT 103	MSH 371	PB1879	PSP128 x PP108	Bayer Biosciences Pvt. Ltd.
**4**	SHT 104	MSH 372	PB1966	PSP83 x PP130	Bayer Biosciences Pvt. Ltd.
**5**	SHT 105	MSH 377	86M22	M461F x M458R	Corteva Agriscince, Hyderabad
**6**	SHT 106	MP 7366 (Check)	-	-	Metahelix Life Science Ltd.
**7**	SHT 107	MSH 378	PB2119607	M453F x M458R	Corteva Agriscince, Hyderabad
**8**	SHT 108	MSH 379	PB2110622	M508F x 9PEZA76R	Corteva Agriscince, Hyderabad
**9**	SHT 109	MSH 380	PB211A114	M453F x 9PNQR68R	Corteva Agriscince, Hyderabad
**10**	SHT 110	MSH 381	PB211A128	M500F x 9PEZA76R	Corteva Agriscince, Hyderabad
**11**	SHT 111	Nandi 75 (Check)	-	-	Nandi Seeds Pvt. Ltd.
**12**	SHT 112	MSH 382	GHB 1288	ICMA1 15777 x J-2635	ICAR-AICRP-PM, JAU, Jamnagar
**13**	SHT 113	MSH 383	GHB 1322	ICMA1 14333 x J-2637	ICAR-AICRP-PM, JAU, Jamnagar
**14**	SHT 114	MSH 384	GHB 1333	JMSA1 20191 x J-2636	ICAR-AICRP-PM, JAU, Jamnagar
**15**	SHT 115	86M64 (Check)	-	-	Pioneer Overseas Corp.
**16**	SHT 116	MSH 385	KPH6310	KP19SA018 x KP19SR020	Kaveri Seeds Co. Ltd.
**17**	SHT 117	MSH 386	VNR 3535	VNRF-14 x VNRBB-1166	VNR Seeds Pvt. Ltd.
**18**	SHT 118	MSH 387	Mahabeej-1005	MBJ-2A x MBJ-1R	MSSCL, Akola
**19**	SHT 119	Proagro 9444 (Check)	-	-	Bayer Biosciences Pvt. Ltd.
**20**	SHT 120	MSH 388	HYMH 4069	HYMA 557 x HYMR 6444	Hi-Yield Agri-Genetics Pvt. Ltd.
**21**	SHT 121	MSH 389	PB1918	PSP99 x PP119	Bayer Biosciences Pvt. Ltd.

Table S2: Experimental details

Code	Name of location	Sowing date	Spacing<br>No. of rows × spacing (m) × row length (m)	Plot size (m^2)
E1	Mandor	18/02/21	6 × 0.60 × 4.0	14.40
E2	Jamnagar	16/02/21	6 × 0.60 × 4.0	14.40
E3	S K Nagar	25/02/21	6×0.45 × 4.0	10.80
E4	Ahmedabad	02/03/21	6 × 0.50×4.0	12.00
E5	NARP, Aurangabad	17/02/21	6 × 0.50 × 4.0	12.00
E6	Coimbatore	26/02/21	6 × 0.50 × 4.0	12.00

Table S3: Mean and range of quantitative traits of pearl millet hybrid at six environments in summer-2021

Traits	E1: Mandor			E2: Jamnagar			E3: S. K. Nagar			E4: Ahmedabad			E5: NARP Aurangabad			E6: Coimbatore		
	Min	Max	Mean	Min	Max	Mean	Min	Max	Mean	Min	Max	Mean	Min	Max	Mean	Min	Max	Mean
DF	44.00	67.00	59.76	44.00	53.00	49.00	49.00	68.00	57.08	45.00	61.00	53.25	57.00	69.00	62.60	42.00	59.00	51.25
DM	79.00	99.00	89.91	74.00	84.00	79.16	80.00	90.00	83.91	74.00	89.00	81.71	92.00	102.00	97.27	83.00	99.00	92.59
PH	153.00	203.00	181.84	172.00	247.00	199.00	181.00	255.00	215.00	160.00	225.00	199.91	115.00	182.00	156.43	168.00	229.00	206.08
PTPP	1.40	4.60	2.84	2.00	4.20	2.76	1.40	2.00	1.72	1.00	2.00	1.16	1.00	4.00	2.03	2.20	3.20	2.53
PL	18.00	31.00	25.44	25.00	34.00	28.71	23.00	31.00	26.89	21.00	35.00	26.86	23.00	32.00	26.05	23.00	33.00	27.81
PD	2.50	3.60	3.11	2.60	3.90	3.26	2.80	3.90	3.32	2.40	3.70	3.07	3.10	3.90	3.31	2.80	4.20	3.55
TSW	7.70	11.60	9.22	8.80	12.50	10.08	8.50	12.80	10.28	4.00	6.80	5.32	9.80	13.70	11.84	9.20	13.80	11.50
PP	145.00	227.00	194.14	163.00	236.00	218.21	171.00	269.00	220.67	165.00	229.00	205.06	145.00	156.00	152.35	90.00	124.00	109.16
SS%	10.00	97.00	75.76	40.00	95.00	70.11	11.00	81.00	54.87	44.00	89.00	73.76	95.00	100.00	98.73	24.00	94.00	79.16
GY	3.28	7.33	5.24	4.81	9.13	7.11	3.80	8.40	6.70	4.47	7.35	6.21	1.90	5.40	3.91	2.96	7.14	4.68
DFY	12.30	26.00	19.46	8.40	15.80	11.77	7.00	12.00	9.60	13.45	24.39	19.37	7.00	11.50	9.70	6.33	13.01	9.52
Fe	20.00	53.00	36.14	39.00	82.00	51.81	45.00	101.00	68.79	26.00	67.00	40.29	51.00	98.00	74.16	36.00	76.00	51.73
Zn	24.00	49.00	34.19	31.00	48.00	39.41	16.00	33.00	22.43	3.00	23.00	10.70	33.00	60.00	42.57	22.00	58.00	42.43

DF: days to 50% flowering, DM: days to maturity, PH: plant height, PTPP: number of productive tillers per plant, PL: panicle length, PD, panicle diameter, TSW: 1000 seed weight, PP: plant population at harvest, SS%: seed set under bagging condition, GY: grain yield, DFY: dry fodder yield, Fe: grain iron content, Zn: grain zinc content

Table S4: location vise mean performance of all 21 pearl millet hybrids in summer-2021

E1: Mandor													
Hybrid	DF	DM	PH	PTPP	PL	PD	TSW	PP	SS	GY	DFY	Fe	Zn
SHT 101	59.33	90.33	193.00	2.47	26.67	3.03	9.17	209.00	55.00	5.06	22.57	39.67	35.33
SHT 102	61.67	91.33	170.00	2.47	29.33	3.13	10.03	191.67	49.00	5.73	17.24	22.67	27.00
SHT 103	59.33	89.00	182.33	3.07	24.67	2.87	10.57	197.00	30.33	4.94	23.03	35.33	31.33
SHT 104	58.00	88.00	189.67	3.13	25.00	2.93	9.60	203.00	93.67	5.19	22.33	32.00	33.33
SHT 105	58.33	88.67	178.00	2.20	25.67	3.13	10.03	193.33	88.00	5.58	21.40	36.67	41.00
SHT 106	62.00	91.67	178.67	2.67	26.00	3.17	9.77	205.67	63.33	5.10	19.32	41.00	38.00
SHT 107	58.33	88.67	192.00	2.87	24.67	3.20	9.10	192.33	87.67	5.62	19.15	34.33	32.33
SHT 108	58.67	89.33	186.33	3.13	27.67	3.37	8.33	201.00	91.67	6.65	19.59	35.67	35.00
SHT 109	63.00	92.33	191.67	3.27	25.00	3.23	8.53	199.00	83.33	5.74	23.90	37.67	30.67
SHT 110	59.00	89.00	190.00	3.07	27.00	3.23	9.03	197.67	85.33	5.99	21.48	37.00	31.33
SHT 111	59.67	90.33	186.33	3.40	25.00	3.13	9.37	197.67	78.00	5.04	20.10	37.67	33.33
SHT 112	59.00	89.00	158.00	3.13	26.33	3.13	8.60	188.67	84.00	4.77	16.10	37.00	33.33
SHT 113	61.00	90.00	187.00	2.53	28.00	3.17	9.73	198.67	83.67	4.78	18.84	38.33	37.67
SHT 114	59.67	89.33	171.67	2.87	24.67	3.17	8.93	186.33	80.67	4.45	13.76	38.33	35.00
SHT 115	66.33	97.33	177.67	2.80	25.67	3.27	8.63	186.00	93.33	5.10	17.42	34.67	36.00
SHT 116	60.00	89.33	173.33	2.67	26.00	3.07	8.67	187.33	89.33	4.90	17.23	40.33	37.67
SHT 117	64.00	92.67	184.00	2.20	25.33	2.90	8.97	188.67	93.33	4.91	17.87	40.00	33.67
SHT 118	44.67	80.33	163.33	2.93	20.00	3.10	10.77	161.33	16.67	3.60	15.62	34.00	33.33
SHT 119	64.67	92.33	194.00	3.67	24.00	2.90	8.10	196.00	80.67	6.01	22.83	31.00	32.00
SHT 120	58.00	89.00	195.33	2.47	25.33	3.00	9.83	189.67	69.67	5.23	20.28	36.00	32.67
SHT 121	60.33	90.00	176.33	2.67	22.33	3.13	7.90	207.00	94.33	5.70	18.60	39.67	38.00
E2: Jamnagar													
Hybrid	DF	DM	PH	PTPP	PL	PD	TSW	PP	SS	GY	DFY	Fe	Zn
SHT 101	47.33	78.00	210.67	2.67	27.67	3.37	9.90	221.33	61.00	6.15	11.40	48.33	39.33
SHT 102	47.33	78.00	185.67	2.47	31.00	3.13	11.70	216.00	52.33	7.62	10.30	43.33	37.67
SHT 103	48.00	77.67	191.67	2.93	25.67	2.80	10.00	221.00	66.00	7.18	13.10	46.33	36.00
SHT 104	47.67	78.00	207.67	2.87	29.33	3.27	11.17	200.67	82.67	7.05	12.77	49.00	40.33

SHT 105	49.33	78.67	232.00	2.33	28.00	3.73	10.43	197.67	76.33	7.61	12.30	53.33	41.33
SHT 106	49.33	79.33	194.00	2.93	28.00	3.10	11.27	214.67	59.00	8.45	14.23	56.00	38.67
SHT 107	49.00	80.00	208.67	2.53	25.33	3.53	10.73	217.00	64.33	8.31	14.03	51.33	35.33
SHT 108	48.33	78.33	201.00	2.67	31.33	3.33	9.10	222.33	80.67	7.71	11.43	50.00	38.67
SHT 109	49.00	78.00	203.33	2.67	26.33	3.10	9.23	225.67	67.00	7.42	10.23	49.67	38.67
SHT 110	49.00	80.00	206.00	2.67	31.67	3.33	10.03	213.67	78.00	8.17	11.23	48.00	38.33
SHT 111	49.67	79.67	203.33	2.47	25.67	3.17	9.87	221.33	60.33	6.44	11.83	46.67	39.67
SHT 112	46.67	76.67	176.67	3.47	28.67	2.93	9.37	230.67	64.00	6.50	10.97	59.33	41.67
SHT 113	50.00	79.67	206.67	2.47	32.33	3.40	10.30	210.00	75.33	6.25	11.57	57.00	39.67
SHT 114	53.00	82.67	199.33	2.47	29.00	3.73	9.97	228.67	84.33	7.54	13.57	66.33	39.00
SHT 115	49.33	80.00	197.67	2.67	30.33	3.53	10.00	214.33	74.67	7.57	12.60	68.33	43.00
SHT 116	49.67	80.33	192.00	2.47	28.67	3.13	9.37	215.00	79.67	6.20	9.40	52.67	42.00
SHT 117	53.00	83.33	200.00	2.80	32.00	3.27	9.37	224.33	79.33	7.65	13.23	47.00	38.67
SHT 118	44.67	75.00	186.33	3.13	28.00	3.50	11.40	221.67	42.67	6.53	11.17	49.33	40.00
SHT 119	49.00	79.67	191.00	3.80	26.33	2.77	9.17	227.33	63.67	5.44	11.00	45.67	38.67
SHT 120	48.67	79.00	195.67	2.67	30.33	3.07	10.27	215.67	75.00	6.63	10.23	47.67	38.67
SHT 121	51.00	80.33	189.67	2.80	27.33	3.20	9.03	223.33	86.00	6.87	10.60	52.67	42.33
E3: S. K. Nagar													
Hybrid	DF	DM	PH	PTPP	PL	PD	TSW	PP	SS	GY	DFY	Fe	Zn
SHT 101	56.33	82.67	214.67	1.53	27.00	3.23	10.10	224.33	47.33	6.23	9.67	70.00	21.67
SHT 102	57.00	82.33	184.67	1.73	27.33	3.20	9.20	210.67	56.67	7.20	9.50	79.33	23.67
SHT 103	56.67	81.00	213.00	1.87	26.67	3.23	10.03	230.00	36.00	7.10	10.33	47.00	20.33
SHT 104	53.33	81.67	219.67	1.87	27.33	3.07	10.57	208.33	40.33	6.23	11.50	60.33	20.00
SHT 105	61.67	88.00	208.00	1.73	26.33	3.43	9.37	225.00	57.00	7.63	10.17	71.33	20.33
SHT 106	57.00	82.33	212.00	1.80	25.33	3.17	10.80	218.33	60.00	6.87	8.33	72.33	27.33
SHT 107	57.00	83.00	240.00	1.73	26.00	3.43	10.47	208.00	52.00	7.40	11.00	71.33	21.33
SHT 108	57.67	81.00	252.67	1.80	29.67	3.63	9.33	231.00	62.00	7.63	10.17	71.00	20.33
SHT 109	58.33	80.33	221.00	1.80	25.33	3.20	10.37	224.00	66.33	6.40	9.17	74.00	19.67
SHT 110	58.33	82.00	236.33	1.67	29.33	3.77	9.33	206.67	72.33	7.93	9.83	59.00	19.33
SHT 111	56.33	82.00	252.33	1.67	27.67	3.40	11.10	207.67	49.00	6.37	10.67	68.33	25.33

SHT 112	58.00	87.33	188.33	1.67	23.33	2.93	10.13	231.67	59.67	6.53	8.50	74.67	24.00
SHT 113	59.67	87.67	236.00	1.67	26.33	3.23	10.60	219.33	60.33	5.57	9.17	92.67	23.67
SHT 114	59.67	89.00	187.33	1.47	28.00	3.30	9.33	235.67	73.67	6.47	8.67	76.00	19.33
SHT 115	55.33	82.67	201.33	1.80	25.00	3.53	10.97	194.33	67.33	6.70	9.67	75.67	24.00
SHT 116	57.33	88.00	213.33	1.87	25.67	3.37	11.00	217.67	53.33	7.10	10.17	64.00	20.67
SHT 117	59.67	86.33	213.33	1.80	27.67	2.87	9.57	234.33	66.33	5.83	10.33	62.00	27.67
SHT 118	52.67	82.67	182.33	1.60	28.00	3.90	12.50	219.33	16.67	4.40	9.17	86.00	26.33
SHT 119	54.00	82.00	217.33	1.53	26.33	3.07	9.80	228.00	43.33	6.50	7.33	56.00	21.33
SHT 120	55.00	83.33	211.33	1.87	28.33	3.27	10.80	227.33	46.00	6.90	9.17	55.00	24.67
SHT 121	57.67	86.67	210.00	1.60	28.00	3.57	10.53	232.33	66.67	7.60	9.17	58.67	20.00
E4: Ahmedabad													
Hybrid	DF	DM	PH	PTPP	PL	PD	TSW	PP	SS	GY	DFY	Fe	Zn
SHT 101	53.00	81.67	201.67	1.00	25.33	3.00	6.20	211.33	81.67	6.74	17.49	35.67	11.33
SHT 102	52.33	80.33	190.00	1.00	34.00	2.90	6.00	191.00	66.67	6.42	16.85	31.33	10.67
SHT 103	51.00	78.00	191.67	1.00	22.67	2.73	4.50	218.33	55.67	6.42	20.52	35.00	5.67
SHT 104	51.00	79.67	211.67	1.00	30.00	3.10	5.03	210.00	78.00	6.57	20.21	35.33	9.00
SHT 105	53.67	81.67	200.67	1.33	27.67	3.10	5.37	201.67	52.00	6.42	16.52	51.67	11.00
SHT 106	53.00	81.33	196.67	1.33	26.67	3.00	6.07	211.33	74.33	6.70	18.56	42.67	8.67
SHT 107	52.67	80.67	215.67	1.00	25.33	3.40	5.57	203.33	78.00	6.63	19.55	41.67	6.67
SHT 108	53.67	82.33	213.33	1.33	30.67	2.77	4.30	201.67	67.00	6.78	21.91	45.67	11.67
SHT 109	54.33	83.00	211.67	1.33	26.67	3.23	4.83	205.00	78.00	6.67	20.56	43.67	15.67
SHT 110	54.33	84.33	201.67	1.00	30.00	3.00	5.60	200.33	67.00	6.83	18.58	45.00	14.67
SHT 111	54.67	83.00	206.67	1.67	25.33	3.20	4.77	188.00	78.00	6.18	17.48	37.00	10.00
SHT 112	53.33	82.67	191.67	1.00	24.33	2.57	5.23	215.00	70.67	5.49	15.04	36.00	8.33
SHT 113	54.33	82.67	206.67	1.33	27.67	3.27	5.77	193.33	74.33	5.95	20.45	40.67	8.33
SHT 114	60.00	88.33	198.33	1.33	24.00	3.60	4.90	213.00	78.00	5.41	23.69	53.33	13.00
SHT 115	51.67	81.00	210.67	1.00	25.67	3.10	5.50	216.67	78.00	6.40	21.52	42.33	12.33
SHT 116	55.33	83.67	196.00	1.00	26.33	2.87	5.77	209.67	85.33	5.86	17.43	44.00	13.67
SHT 117	55.00	83.33	206.67	1.33	29.67	3.10	4.97	207.00	78.00	5.80	21.09	40.33	10.67

SHT 118	46.67	75.00	171.67	1.00	26.00	3.60	5.87	192.67	67.00	5.08	19.31	34.00	9.33
SHT 119	52.33	81.00	195.00	1.33	21.67	2.70	4.73	215.33	78.00	5.32	19.01	39.33	10.67
SHT 120	52.67	80.33	191.67	1.00	29.67	2.80	5.63	208.00	85.33	6.20	23.48	37.33	10.00
SHT 121	53.33	82.00	188.33	1.00	24.67	3.37	5.17	193.67	78.00	6.50	17.53	34.00	13.33
E5: NARP													
Hybrid	DF	DM	PH	PTPP	PL	PD	TSW	PP	SS	GY	DFY	Fe	Zn
SHT 101	60.00	94.00	157.00	1.33	24.00	3.40	11.90	150.67	99.67	3.23	9.90	58.33	35.00
SHT 102	61.00	96.00	136.00	2.00	31.33	3.20	11.23	153.67	98.67	3.90	8.50	65.67	44.00
SHT 103	58.33	93.33	159.00	2.00	24.33	3.10	10.57	152.00	97.00	4.93	9.63	57.33	43.67
SHT 104	59.00	94.33	163.33	1.67	24.67	3.27	12.23	150.00	99.00	2.98	10.13	61.67	38.33
SHT 105	66.00	100.00	155.33	1.67	24.67	3.23	12.40	153.33	98.33	3.16	10.03	80.33	49.33
SHT 106	65.00	97.67	161.67	2.67	27.00	3.20	13.07	154.33	99.67	4.25	9.90	83.67	45.67
SHT 107	64.00	96.67	163.67	2.00	23.67	3.43	11.93	152.67	99.67	2.62	10.90	96.33	47.00
SHT 108	60.33	97.00	167.00	2.67	28.33	3.47	10.03	155.00	99.33	3.88	10.33	89.00	52.33
SHT 109	65.00	98.67	162.67	2.33	25.00	3.37	11.23	152.00	99.67	4.45	10.63	69.33	38.33
SHT 110	64.00	96.33	140.67	1.67	27.00	3.20	10.90	152.00	98.33	2.73	9.67	73.00	44.00
SHT 111	63.00	98.00	175.00	2.33	24.33	3.37	11.10	153.33	99.00	4.09	9.63	63.00	41.33
SHT 112	61.33	94.67	159.67	2.67	26.33	3.13	11.90	150.67	96.00	5.02	10.03	73.33	41.67
SHT 113	61.33	96.33	155.33	2.00	28.67	3.37	13.27	150.67	97.67	3.65	9.47	82.33	44.67
SHT 114	68.00	101.00	160.00	2.00	24.67	3.63	11.40	154.00	99.67	3.96	10.53	89.33	46.33
SHT 115	65.00	99.33	165.33	2.33	26.33	3.70	12.13	154.67	99.00	2.03	7.60	88.00	48.00
SHT 116	63.00	99.00	148.67	2.00	24.67	3.27	13.33	150.67	100.00	4.22	10.50	55.67	36.33
SHT 117	63.00	100.00	154.00	1.00	28.00	3.20	12.93	151.33	98.67	4.35	9.77	70.67	37.33
SHT 118	62.00	96.33	122.00	2.33	26.00	3.20	12.27	150.67	97.00	3.88	9.30	84.00	42.33
SHT 119	62.67	98.00	160.67	1.67	25.00	3.13	10.53	153.00	98.00	5.12	10.13	73.67	43.67
SHT 120	62.00	99.00	163.67	1.67	27.67	3.37	12.70	152.67	99.67	4.72	9.17	67.67	37.00
SHT 121	60.67	97.00	154.33	2.67	25.33	3.17	11.47	152.00	99.33	4.99	8.00	75.00	37.67
E6: Coimbatore													
Hybrid	DF	DM	PH	PTPP	PL	PD	TSW	PP	SS	GY	DFY	Fe	Zn
SHT 101	53.00	94.00	201.33	2.53	25.67	3.47	12.97	102.00	77.00	4.30	8.88	48.33	44.67

SHT 102	49.67	91.33	175.67	2.67	30.33	3.43	13.10	115.33	84.33	4.51	8.70	59.00	46.00
SHT 103	51.33	92.00	205.67	2.40	23.00	3.03	11.13	107.00	65.67	4.79	10.28	40.00	37.67
SHT 104	49.00	90.33	215.67	2.67	28.67	3.37	12.20	110.67	87.33	5.33	11.32	50.33	39.00
SHT 105	50.67	93.00	200.00	2.47	26.00	3.77	12.93	117.00	49.33	5.99	10.97	56.33	45.33
SHT 106	48.67	90.67	206.33	2.67	24.67	3.43	13.03	121.00	86.67	5.79	10.09	55.00	38.67
SHT 107	50.33	92.00	210.33	2.27	25.67	3.77	10.60	112.67	85.00	4.57	8.90	56.00	42.33
SHT 108	50.67	93.00	213.33	2.67	29.33	3.60	9.77	105.67	83.67	4.43	9.89	49.00	41.67
SHT 109	53.33	95.67	211.33	2.53	27.00	3.63	9.73	108.67	90.33	5.96	10.37	44.33	39.67
SHT 110	51.33	92.67	215.67	2.67	32.67	4.00	11.03	113.33	79.67	5.22	11.35	48.33	38.67
SHT 111	54.00	95.00	214.67	2.53	26.33	3.40	11.13	104.67	75.00	5.53	11.11	38.00	33.67
SHT 112	51.67	92.67	201.00	2.40	27.00	3.13	10.10	108.00	83.33	4.14	9.84	52.00	43.33
SHT 113	49.00	91.00	210.00	2.47	30.67	3.73	12.83	109.67	89.33	3.64	9.08	61.00	51.33
SHT 114	58.00	97.00	200.33	2.40	27.33	4.10	11.10	101.33	92.33	4.18	9.98	72.67	47.67
SHT 115	47.67	89.00	210.33	2.53	28.33	3.63	12.97	106.67	88.67	6.77	11.58	65.33	53.00
SHT 116	53.33	92.67	179.67	2.40	30.33	3.40	10.10	106.00	71.00	4.23	8.09	49.00	42.33
SHT 117	53.67	94.00	203.00	2.40	30.33	3.50	11.17	96.00	89.00	3.48	7.62	54.00	39.00
SHT 118	42.67	84.67	213.67	2.60	28.33	3.80	12.20	106.67	29.33	4.05	7.93	50.00	41.67
SHT 119	55.67	96.67	223.00	2.87	25.33	3.20	10.07	118.00	82.33	3.39	8.48	44.33	38.00
SHT 120	48.67	90.33	207.00	2.40	32.00	3.73	12.20	110.67	83.67	3.89	7.59	45.00	45.33
SHT 121	54.00	96.67	209.67	2.53	25.00	3.50	11.10	111.33	89.33	4.14	7.95	48.33	42.00

DF: days to 50% flowering, DM: days to maturity, PH: plant height, PTPP: number of productive tillers per plant, PL: panicle length, PD, panicle diameter, TSW: 1000 seed weight, PP: plant population at harvest, SS%: seed set under bagging condition, GY: grain yield, DFY: dry fodder yield, Fe: grain iron content, Zn: grain zinc content.

Table S5: PC value, mean over environment and WAAS score for dry fodder yield, Fe content and Zn content of 21 hybrids evaluated over six different locations

Hybrid	Mean over environment	PC1	PC2	PC3	PC4	PC5	WAAS
**1. Dry fodder yield**							
SHT 101	13.318	–1.040	0.046	0.308	0.312	0.124	0.608
SHT 102	11.848	0.025	–0.581	0.173	–0.383	0.284	0.228
SHT 103	14.483	–0.574	0.475	–0.353	0.374	0.419	0.506
SHT 104	14.709	–0.470	0.070	–0.263	–0.214	0.457	0.324
SHT 105	13.565	–0.871	–0.751	–0.222	0.086	0.024	0.684
SHT 106	13.405	0.055	–0.430	–0.544	1.079	–0.328	0.286
SHT 107	13.922	0.289	–0.353	0.539	0.703	0.406	0.364
SHT 108	13.888	0.352	0.541	0.099	–0.437	–0.123	0.376
SHT 109	14.144	–0.968	0.913	–0.044	–0.416	–0.623	0.816
SHT 110	13.691	–0.652	–0.114	–0.467	–0.450	–0.153	0.45
SHT 111	13.469	–0.445	–0.676	–0.251	–0.364	0.216	0.474
SHT 112	11.747	0.049	–1.403	0.103	–0.134	–0.700	0.461
SHT 113	13.095	0.297	0.313	–0.020	–0.009	–0.062	0.245
SHT 114	13.366	2.130	0.104	–0.306	0.125	–0.706	1.206
SHT 115	13.397	0.828	0.061	–1.307	–0.446	0.446	0.615
SHT 116	12.136	0.094	–0.473	1.089	–0.712	–0.175	0.332
SHT 117	13.319	0.769	0.230	0.533	0.605	0.598	0.582
SHT 118	12.082	0.861	–0.206	0.416	–0.072	0.023	0.546
SHT 119	13.131	–0.913	0.744	0.063	0.627	–0.767	0.765
SHT 120	13.321	0.354	1.595	0.333	–0.270	0.128	0.673
SHT 121	11.975	–0.171	–0.105	0.121	–0.004	0.513	0.154
E1	19.460	–2.781	1.141	–0.086	0.301	0.118	1.790
E2	11.771	0.792	–0.848	–0.474	1.629	0.394	0.819
E3	9.603	0.137	–0.778	0.847	–0.828	1.294	0.480
E4	19.371	1.842	2.117	–0.180	–0.253	–0.033	1.569
E5	9.703	0.132	–0.582	1.333	0.083	–1.247	0.411
E6	9.523	–0.122	–1.049	–1.440	–0.932	–0.525	0.567
**2. Fe content**							
SHT 101	50.056	–0.982	2.185	0.153	–0.225	–0.757	1.249
SHT 102	50.222	2.237	2.090	–1.675	1.340	–0.858	2.095
SHT 103	43.500	–2.620	–0.358	–0.215	–0.569	–0.058	1.463
SHT 104	48.111	–0.886	0.577	–1.196	–0.058	–0.170	0.833

SHT 105	58.278	–0.029	–0.667	–0.032	1.771	0.764	0.244
SHT 106	58.444	0.352	–0.664	0.397	–0.549	–0.330	0.464
**Hybrid**	**Mean over environment**	**PC1**	**PC2**	**PC3**	**PC4**	**PC5**	**WAAS**
SHT 107	58.500	1.901	–2.439	0.590	0.068	–0.694	1.865
SHT 108	56.722	0.955	–1.703	1.501	0.716	0.454	1.296
SHT 109	53.111	–0.280	1.052	1.473	0.666	1.076	0.736
SHT 110	51.722	–1.172	–1.052	0.294	0.971	–0.049	0.987
SHT 111	48.444	–0.817	1.286	1.810	–0.266	0.660	1.139
SHT 112	55.389	0.396	0.883	–0.245	–1.706	0.818	0.534
SHT 113	62.000	2.327	1.749	0.272	–0.151	–0.144	1.794
SHT 114	66.000	0.636	–1.080	–2.374	0.695	0.589	1.072
SHT 115	62.389	1.280	–0.842	–2.027	–1.522	1.261	1.256
SHT 116	50.944	–2.304	1.703	–0.478	0.513	0.604	1.800
SHT 117	52.333	–0.952	–0.236	–0.288	0.509	–1.824	0.602
SHT 118	56.222	2.679	0.763	1.610	–0.587	–0.278	1.858
SHT 119	48.333	–0.552	–1.509	0.294	0.475	0.264	0.831
SHT 120	48.111	–1.392	–0.726	0.061	–0.302	–0.359	0.948
SHT 121	51.389	–0.777	–1.012	0.075	–1.791	–0.969	0.740
E1	36.143	–3.621	0.821	2.080	–1.158	–1.798	2.426
E2	51.810	–1.230	0.125	–1.616	–2.522	1.999	0.923
E3	68.794	3.636	3.968	1.454	0.340	0.364	3.387
E4	40.286	–2.538	–0.796	0.527	2.999	1.340	1.620
E5	74.159	3.082	–4.377	1.342	–0.478	–0.289	3.230
E6	51.730	0.671	0.258	–3.787	0.819	–1.617	1.047
**3. Zn content**							
SHT 101	31.222	–1.377	1.076	–0.016	0.295	–0.496	0.992
SHT 102	31.500	0.687	0.678	0.842	–1.869	0.189	0.716
SHT 103	29.111	0.819	–0.703	0.307	0.270	0.230	0.673
SHT 104	30.000	–0.689	–0.153	–0.300	0.351	0.925	0.428
SHT 105	34.722	1.090	0.114	–0.941	1.467	–0.015	0.732
SHT 106	32.833	0.246	–1.421	0.887	1.011	–0.706	0.773
SHT 107	30.833	1.485	–0.096	0.476	0.119	–0.454	0.809
SHT 108	33.278	1.859	–0.769	–0.882	–0.047	–0.106	1.289
SHT 109	30.444	–0.914	–0.085	–1.122	–1.345	–0.382	0.679
SHT 110	31.056	0.195	–0.685	–1.260	–1.106	–0.241	0.581
SHT 111	30.556	–0.619	–1.940	0.361	0.011	0.319	1.008
SHT 112	32.056	–0.169	0.178	0.718	0.123	1.182	0.286
SHT 113	34.222	0.654	1.598	0.786	0.884	–0.325	0.998

SHT 114	33.389	0.881	0.989	–0.861	–0.233	–0.442	0.913
SHT 115	36.056	0.886	1.556	0.249	–0.218	0.567	0.978
**Hybrid**	**Mean over environment**	**PC1**	**PC2**	**PC3**	**PC4**	**PC5**	**WAAS**
SHT 116	32.111	–1.567	0.440	–0.923	0.580	0.009	1.055
SHT 117	31.167	–1.303	–0.715	1.120	–0.182	–0.664	1.068
SHT 118	32.167	–0.189	–0.413	1.016	–0.134	0.265	0.436
SHT 119	30.722	0.238	–0.876	–0.323	–0.384	0.275	0.47
SHT 120	31.389	–0.911	0.906	0.956	–0.318	–0.370	0.919
SHT 121	32.222	–1.303	0.320	–1.091	0.727	0.240	0.929
E1	34.190	–0.719	–0.290	–0.746	2.827	–0.710	0.581
E2	39.413	–1.291	–0.205	–0.435	0.140	1.918	0.748
E3	22.429	–1.082	–1.404	2.897	–0.548	–0.402	1.568
E4	10.698	–1.425	–0.425	–2.033	–1.901	–0.793	1.216
E5	42.571	3.821	–1.347	–0.346	–0.233	0.132	2.267
E6	42.429	0.695	3.670	0.662	–0.285	–0.146	1.686

PC: principal component, WAAS: weighted average of absolute score, E1: Mandor, E2: Jamnagar, E3: S. K. Nagar, E4: Ahmedabad, E5: NARP, Aurangabad, E6: Coimbatore.

Table S6: Pearson's correlation coefficient among all traits over environments

	DF	DM	PH	NPT	PL	PD	TSW	PP	SS	GY	DFY	Fe	Zn
**DF**	1.00												
**DM**	0.68	1.00											
**PH**	–0.44	–0.48	1.00										
**NPT**	–0.07	0.14	–0.09	1.00									
**PL**	–0.29	–0.20	0.19	0.02	1.00								
**PD**	–0.07	0.21	0.16	0.08	0.23	1.00							
**TSW**	0.11	0.46	–0.21	0.38	0.05	0.40	1.00						
**PP**	–0.03	–0.66	0.19	–0.14	0.00	–0.37	–0.45	1.00					
**SS**	0.38	0.54	–0.39	0.06	–0.02	0.06	0.10	–0.36	1.00				
**GY**	–0.36	–0.68	0.53	–0.07	0.22	–0.09	–0.32	0.60	–0.35	1.00			
**DFY**	0.06	–0.23	0.02	–0.08	–0.15	–0.38	–0.68	0.32	–0.03	0.20	1.00		
**Fe**	0.30	0.30	–0.15	–0.08	0.02	0.33	0.50	–0.10	0.14	–0.18	–0.62	1.00	
**Zn**	0.06	0.49	–0.33	0.59	0.08	0.33	0.74	–0.50	0.34	–0.40	–0.46	0.32	1.00

DF: days to 50% flowering, DM: days to maturity, PH: plant height, NPT: number of productive tillers per plant, PL: panicle length, PD: panicle diameter, TSW: 1000 seed weight, PP: plant population at harvest, SS: seed set under bagging condition, GY: grain yield, DFY: dry fodder yield, Fe: grain iron content and Zn: grain zinc content

Table S7: Genotype-ideotype (ID) scores, MTSI values for the 21 pearl millet hybrids for the first four factors along with relative contribution of each factor towards the MTSI

Sr. No.	**Hybrid**	**FA1**	**RC %**	**FA2**	**RC %**	**FA3**	**RC %**	**FA4**	**RC %**	**MTSI**
1	SHT 101	–4.000	34.92	–3.715	32.43	–2.770	24.18	–0.971	8.48	6.208
2	SHT 102	–3.917	47.28	–2.882	34.79	–1.468	17.72	–0.017	0.21	7.393
3	SHT 103	–5.045	50.24	–2.841	28.29	–1.796	17.88	0.360	3.58	6.795
4	SHT 104	–5.746	39.06	–4.655	31.64	–4.213	28.64	–0.097	0.66	**3.853**
5	SHT 105	–3.162	26.5	–4.925	41.27	–3.142	26.33	0.705	5.91	5.533
6	SHT 106	–4.264	30.27	–5.095	36.17	–4.464	31.69	–0.263	1.87	**4.153**
7	SHT 107	–4.575	33.23	–3.948	28.68	–3.504	25.45	–1.740	12.64	5.697
8	SHT 108	–4.851	37.74	–5.797	45.10	–1.670	12.99	0.537	4.18	5.37
9	SHT 109	–3.295	29.25	–5.315	47.19	–1.801	15.99	–0.853	7.57	6.384
10	SHT 110	–4.420	34.42	–5.072	39.5	–2.835	22.08	0.514	4.00	**4.931**
11	SHT 111	–3.823	33.11	–3.886	33.66	–2.289	19.83	–1.548	13.41	6.656
12	SHT 112	–3.598	25.45	–4.788	33.86	–2.930	20.72	2.823	19.97	5.825
13	SHT 113	–3.920	28.58	–4.811	35.08	–3.978	29.00	–1.007	7.34	4.977
14	SHT 114	–1.484	15.63	–4.873	51.33	–3.049	32.12	0.087	0.92	6.865
15	SHT 115	–1.982	16.92	–4.681	39.97	–3.956	33.78	–1.092	9.32	6.401
16	SHT 116	–3.312	37.98	–3.736	42.84	–1.270	14.56	–0.403	4.62	7.351
17	SHT 117	–3.750	30.79	–5.485	45.03	–2.645	21.71	0.301	2.47	5.258
18	SHT 118	–3.343	31.59	–1.603	15.15	–4.568	43.16	1.069	10.10	7.129
19	SHT 119	–2.585	26.33	–3.923	39.95	–3.116	31.73	0.195	1.99	6.478
20	SHT 120	–4.693	35.34	–4.643	34.97	–3.817	28.75	–0.125	0.94	4.496
21	SHT 121	–3.332	32.12	–4.292	41.37	–2.307	22.24	–0.443	4.27	6.374

FA: factor analysis, RC: relative contribution and MTSI: multi trait stability index
